# Climate Tolerances and Habitat Requirements Jointly Shape the Elevational Distribution of the American Pika (*Ochotona princeps*), with Implications for Climate Change Effects

**DOI:** 10.1371/journal.pone.0131082

**Published:** 2015-08-05

**Authors:** Leah H. Yandow, Anna D. Chalfoun, Daniel F. Doak

**Affiliations:** 1 Department of Zoology and Physiology, University of Wyoming, 1000 East University Avenue, Laramie, Wyoming, 82071, United States of America; 2 Wyoming Cooperative Fish and Wildlife Research Unit, Department of Zoology and Physiology (3166), University of Wyoming, 1000 East University Avenue, Laramie, Wyoming, 82071, United States of America; 3 U.S. Geological Survey Wyoming Cooperative Fish and Wildlife Research Unit, Department of Zoology and Physiology (3166), University of Wyoming, 1000 East University Avenue, Laramie, Wyoming, 82071, United States of America; 4 Environmental Studies Program, University of Colorado Boulder, 1201 17^th^ St., 397 UCB, Boulder, Colorado, 80309, United States of America; University of Hawaii at Manoa, UNITED STATES

## Abstract

Some of the most compelling examples of ecological responses to climate change are elevational range shifts of individual species, which have been observed throughout the world. A growing body of evidence, however, suggests substantial mediation of simple range shifts due to climate change by other limiting factors. Understanding limiting factors for a species within different contexts, therefore, is critical for predicting responses to climate change. The American pika (*Ochotona princeps*) is an ideal species for investigating distributions in relation to climate because of their unusual and well-understood natural history as well as observed shifts to higher elevation in parts of their range. We tested three hypotheses for the climatic or habitat characteristics that may limit pika presence and abundance: *summer heat*, *winter snowpack*, and *forage availability*. We performed these tests using an index of pika abundance gathered in a region where environmental influences on pika distribution have not been well-characterized. We estimated relative pika abundance via scat surveys and quantified climatic and habitat characteristics across two North-Central Rocky Mountain Ranges, the Wind River and Bighorn ranges in Wyoming, USA. Pika scat density was highest at mid-elevations and increased linearly with forage availability in both ranges. Scat density also increased with temperatures conducive to forage plant growth, and showed a unimodal relationship with the number of days below -5°C, which is modulated by insulating snowpack. Our results provide support for both the forage availability and winter snowpack hypotheses. Especially in montane systems, considering the context-dependent nature of climate effects across regions and elevations as well as interactions between climatic and other critical habitat characteristics, will be essential for predicting future species distributions.

## Introduction

Though climate change is now exceptionally well supported, the continued and future ecological effects of climate change for a wide variety of species are less certain. The most compelling evidence for ecological effects of climate change comes from shifts in species distributions [1–3]. Elevation is frequently used as a surrogate for climate in predicting shifts in range limits, and many researchers have predicted that species will migrate upslope with ongoing climate change, because temperature, and in some cases precipitation, are strongly related to elevation [4, 5]. However, syntheses suggest that many species do not actually fit the general expectations for range shifts [6]. Effects of complex topography and other co-varying habitat characteristics in mountain systems are likely to complicate the relationship between elevation and climatic conditions, and species’ responses to shifting temperatures. Thus, more accurately understanding how and why species are limited by climate and other factors in mountain habitats is an important step in assessing the ecological effects of climate change.

In spite of this complexity, the multitude of alpine species that are responding to climate change demonstrates that alpine environments are some of the most susceptible habitats to changing climate [7–10]. The American pika *(Ochotona princeps*) is often considered one of the most sensitive alpine species due to its unusual natural history, and has been touted as an important indicator of changes in alpine habitats. Pikas are herbivorous lagomorphs that do not migrate or hibernate and maintain a high metabolism through the summer, rendering them vulnerable to the full suite of seasonal climate stressors and especially to temperature extremes [11, 12]. Within habitats of suitable climate conditions, pikas occupy specific substrates that provide refuge from predators and buffer them from thermal changes. These habitats vary across their range and include mine tailings, rocky hills, lava beds and, in the Rocky Mountains, primarily high elevation talus slopes. Since pikas are limited by environmental conditions year-round and highly associated with specific habitats [13], there is strong potential for climate, in combination with other factors, to limit their distribution.

The vulnerability of the American pika to extreme high temperatures has been recognized for decades [12]. However, more recently, a focus on climate change impacts on ecological systems has prompted new research on the severity and ubiquity of these limitations. Pika populations in the Great Basin have shown patterns of extinction associated with high summer temperatures, acute cold stress, forb cover and summer precipitation [11, 14–16]. In contrast, Southern Rocky Mountain populations have shown little or no evidence of decline, but rather show local extirpations associated with consistently dry sites [17]. In other parts of the pika’s range, stable populations have been the norm, including at some exceptionally low elevation sites and across a variety of substrates [18–21]. These patterns suggest context-dependent responses to aspects of climate within a single species across its range. The American pika is therefore an ideal focal species for the investigation of geographic variation in the relative influence of climatic and habitat variables across elevation, to improve understanding of context-dependent effects of climate change.

The most dramatic changes in pika distribution have been documented on their range periphery, in places that are relatively hot, dry, and often at low elevations. What remains unclear is how climatic characteristics affect pikas in more northern, mesic habitats such as within the North-Central Rocky Mountains, where pikas primarily inhabit alpine talus slopes. Hot summer temperatures may be less of a concern at these latitudes, but with increasing year-round temperature and precipitation variability, ambient climatic conditions may still limit pika populations. In particular, in high-elevation montane environments, persistent insulating snow cover is likely to be critical for over-winter survival. A combination of higher frequency of melt-freeze cycles and a lack of insulating snow may lead to ruined haypiles (drying food needed for overwinter survival) and/or acute cold stress [11]. Pikas at more northern latitudes, therefore, may not have the same climate and habitat limitations as those regions in which studies have shown higher occupancy at high elevation and on northeasterly slopes [11, 18, 21]. Moreover, few studies have evaluated the relative abundance of pikas in relation to potential limiting factors, instead using presence/absence data to index local populations (but see 14, 22). Estimates of abundance may provide more nuanced inference about habitat suitability than these occupancy indices.

We analyzed climate-related habitat features (i.e., elevation, aspect, elevation difference to summit) and other habitat variables thought to be important for pika persistence (i.e., talus depth and forage availability) and their joint effects on pika relative abundance as indexed by scat density. We also tested for effects of several derived climate variables on scat density and investigated which of these climate variables are indexed by elevation at our sites. Our primary objectives were to 1) evaluate which habitat features may be most limiting to pikas in the northern part of their range, including both features thought to index some aspect of climate and other local habitat features identified as potentially important for persistence, 2) test the generality of these patterns across two similar yet geographically distinct mountain ranges in the North-Central Rocky Mountains, and 3) use derived climate variables from temperature sensors to investigate which aspects of climate most co-vary with elevation in this region. We first looked for patterns in scat density with elevation and then tested three non-mutually-exclusive hypotheses concerning the factors limiting pika abundance and distribution, each based on previous research and the natural history of the American pika. The *summer heat hypothesis* suggests that high summer temperatures limit pika numbers via acute and chronic heat stress and reduced foraging and caching time [11, 12, 23]. Under this hypothesis we expected low pika abundance at characteristically warmer sites based on habitat features, such as at low elevation and southern aspects. The *winter snowpack hypothesis* emphasizes the importance of an insulating snowpack during winter whereby variable snowpack could lead to cold stress or death either by exposure to extreme cold, higher predation rates, and/or starvation by damaged haypiles [11]. If winter snowpack was influencing pika abundance at our sites, we predicted lower pika abundance at sites prone to variable snowpack conditions such as near the summit, on wind-scoured slopes, or particularly low elevation sites where repeated melt-freeze cycles are more likely to occur. Finally, the *forage availability hypothesis* suggests that food availability has a strong limiting effect on pika numbers and distribution [24–26]. If forage availability was a primary limiting factor on pikas, we expected a positive association between pikas and both forage availability and climatic variables that create favorable plant growth conditions during summer. While environmental conditions may also modify haying behavior, we reasoned that the overall amount of forage available should still influence the numbers of pikas within an area. Overall, our aim was to evaluate the support for these three hypotheses and thereby investigate which factors may be most limiting to pika relative abundance in our region.

## Materials and Methods

### Approach

We selected climatic and habitat variables related to the predictions of our three hypotheses (Table 1) and assessed pika relative abundance within two distinct mountain ranges in Wyoming, USA, using scat density as an index. We tested general linear models including combinations of elevation, aspect, elevation difference to summit, talus depth, and two metrics of forage availability as potential predictive factors (Table 1), and judged the support for each using information criteria (AIC_*c*_). We then fit a series of models that included factors other than elevation in the best model of the first analysis but replaced elevation with climatic variables (days above 15°C, winter mean temperature, days below 0°C, days below -5°C, days below -10°C, days above 10°C, total degree days, length of growing season, and mean summer temperature) derived from the local temperature data from one of the mountain ranges in a (Table 1). Finally, we tested a full model suite of climatic variables and their ability to predict pika scat abundance.

**Table 1:** Habitat variables (top) and derived climate variables (bottom) used in general linear models and model selection analyses of relative pika (*Ochotona princeps*) scat density in the Wind River (2010) and Bighorn (2011) mountain ranges, Wyoming, USA.

**Variable**	**Relationship to Climate**	**Relevant Hypotheses**
Elevation	Inversely related to temperature	Summer heat; winter snowpack
Aspect	South and west are warmer aspects	Summer heat; winter snowpack
Elevation difference to summit	Index of wind and melt/freeze exposure	Summer heat; winter snowpack
Patch forage	N/A	Forage availability
Perimeter forage	N/A	Forage availability
Talus depth	N/A	N/A
**Variable**	**Description**	**Relevant Hypothesis**
days above 15°C	Total of days temperature ≥ 15	Summer heat
winter mean	Average temperature of days < 0	Snowpack
days below 0°C	Total # of days temperature < 0	Snowpack
days below -5°C	Total # of days temperature < -5	Snowpack
days below -10°C	Total # of days temperature < -10	Snowpack
days above 10°C	Total # of days temperature < 10	Forage availability
total degree days	Total # days above 0 x average temperature	Forage availability
length of growing season	Total # of days above 0	Forage availability
summer mean	Average temperature of days > 0	Forage availability

N/A = variables included in models as a necessary component of pika habitat but not considered a potential proxy for climate.

### Study sites

Our sites were located within the Wind River (hereafter Winds) and Bighorn mountain ranges, which are separated by a 200 km-wide basin of sagebrush steppe. We chose the Winds for its variation in topography and climate and abundant alpine habitat. Using aerial imagery, we selected between 30 and 40 potential survey sites in each of four quadrants: northeast, southeast, southwest, and northwest. At least eight of those sites were in each of three elevation classes: < 3,000 m; 3,000–3,500 m; and > 3,500 m. Similarly, we stratified across northeast, southeast, southwest, and northwest aspects, and within each elevation group assigned at least two sites for the four aspects. We used the intercardinal directions because prevailing weather patterns in the Winds are primarily northwesterly and southeasterly. To ensure that potential sites covered a broad range of available climatic conditions, we visually assessed precipitation and maximum and minimum temperatures from Parameter-elevation Regression on Independent Slopes Model (PRISM) data for January and July of each year from 2000–2009, choosing potential sites that covered a broad range of pixel values for each of the climate variables. Similarly, we used National Agricultural Imagery Program (NAIP) aerial imagery from July 2009 to evaluate variation in forage availability and select sites with a wide range of number of red pixels, indicative of vegetation, within and around the potential survey area. We also chose sites with variation in microclimate as indicated by snow presence in the imagery. In our list of potential sites, we included those with both lingering snowfields and early snowmelt, as well as sites with evident patches of vegetation and those with little to no apparent vegetation. We ultimately selected 43 survey sites (i.e., talus patches; elevation 2540–3926 m) in 2010, based on sampling across the broadest range of attributes available and accessibility. We considered a patch of talus or potential survey site to be a rock field primarily consisting of rocks > 0.5 m on their longest axis that were often interspersed with small alpine meadows or other vegetation. We defined the size and boundaries of each survey site based on local features such as a cliff band, a marked change in aspect, or conspicuous line of vegetation such as trees or a meadow. To ensure that we sampled potentially suitable sites, each site used in the analysis had to include at least 75% talus, as ground-truthed in the field. We resurveyed 9 of the 43 sites in 2011 to test for potential year effects. Resurveyed sites included samples from each of the four regions of the range, as well as sites at low, mid, or high elevations, and spanned all aspects.

In 2011, we conducted surveys in our second focal mountain range, the Bighorns. Similar in geology, climate, and wildlife communities, the Bighorns served as an appropriate comparison area to test the generality of patterns observed in the Winds. We chose 60 potential sites in the Bighorns using the same methodology of stratifying survey sites across habitat and climate attributes. Based on field logistics and accessibility, we ultimately surveyed 40 of those sites (2158–3897 m) from 25 June -13 August 2011.

### Ethics Statement

All data collection for our study was purely observational and did not involve a threatened or endangered species and therefore the only permits obtained for this research were for access on the Wind River Reservation for 2 of the 83 survey sites. We obtained permission to access those sites through the Tribal Fish and Game. All other sites in the Winds were located within the Shoshone and Bridger-Teton National Forests (between 12T 593644mE, 4804674mN and 668445mE, 4722213m N; NAD 83) and therefore did not require permission for access. Sites in the Bighorns were all on public land in Bighorn National Forest (between 13T 322689mE, 4927468mN and 344272mE, 4890903mN; NAD 83) and therefore did not require permission for access.

### Pika Relative Abundance

Pikas are typically studied using occupancy methods due to logistical constraints [14]. Presence/absence data alone, however, yield limited ecological inference. We therefore used pika scat density as an index of relative abundance, as a compromise between occupancy and density estimation. Scat counts have been one of the most widely used methods for quantifying the relative abundance of mammalian species [27–31], and occupancy in lagomorphs [17, 31–34] including pikas [22], because they provide a more stable record of presence and habitat use than do visual observations. Pika scat is conspicuous and often persists for multiple years [35]. Additionally, large sample sizes of spatially-independent sites spanning a diversity of climatic and other habitat variation were essential for testing our hypotheses. For broad, multi-site studies like ours, scat surveys are feasible to conduct across a large number of sites within a reasonable amount of time.

We acknowledge several caveats related to our use of scat density for estimating pika numbers. Scat degrades over time, and possibly at an inconsistent rate across sites depending on moisture levels. There is also some error associated with counting scat, as inevitably some piles will not be located. However, we assume that environmental conditions are similar enough across sites and that decomposition rates should also have been similar. We also very carefully attempted to locate all scat piles, and assumed that scat that was unaccounted for was evenly distributed across sites. Direct abundance estimates are indeed ideal for more accurate delineation of proximate abundance and temporal comparisons of abundance. Their usefulness for characterizing factors influencing the distribution of a species known for its metapopulation dynamics and weather-dependent behavior, however, is limited. Methods for assessing pika abundance that rely on aural and/or visual detections, for example, are subject to the vagaries of ambient weather conditions and are time-intensive [14], rendering them impractical for studies necessitating broad-scale spatial replication in remote and widely dispersed survey sites. Similarly, using only very recent indicators of abundance, such as fresh sign, can introduce confounding effects of inter-annual variation in numbers that are, from the point of view of our questions, statistical noise. We therefore concluded that scat density was the best metric for indexing general abundance across several sites within a single year, as a time-averaged signal of local habitat suitability.

We also assumed that pika abundance across years, as indexed by scat density, was a reliable indicator of habitat suitability, a reasonable assumption given pika life history. Pikas are site-faithful and have small home ranges. After dispersal, they occupy a territory for their lifetime without seasonal movement and are therefore susceptible to whatever ambient abiotic and biotic conditions exist at that particular site. With very little movement of individuals post-dispersal we expected that the density of scat, and therefore pikas, in a patch should be a good indicator of how well that patch consistently supported pikas during recent years. Therefore, using scat as an estimate of moderate-term abundance allowed us to infer the suitability of an area for pika persistence, an important consideration for a species that is well-known to show transient extirpations and recolonizations at local sites [36–38]. The method also enabled us to avoid the potential confounding effects of ephemeral weather influences that can skew other indices such as aural or visual pika detections. Even though pikas are typically conspicuous, weather and season strongly influence detections of individuals within a single day or across a season. We also considered using haypiles or only fresh scat as an indicator of pikas. However, as proxies for abundance, these variables are also subject to temporal inconsistency. Haypiles are usually difficult to detect before August and fresh scat is not simple to score with high repeatability (Yandow, pers. obs.), and indexes only current year abundance, thereby ignoring the inter-annual fluctuations in abundance. Haypiles were moderately correlated with pika scat in the Winds in 2010 (*r* = 0.29, N = 43, *P* = 0.05) and strongly correlated with scat in the Bighorns in 2011 (*r* = 0.78, N = 40, *P* < 0.01), providing additional evidence that scat was a reasonable predictor of pika relative abundance.

We sampled scat at each survey site along parallel line transects [33, 34, 39]. We established the starting point for each transect in one of three ways, as dictated by individual field situations: 1) at a talus/vegetation interface; 2) at a distinct natural feature of the landscape (rock outcropping, cliff edge, etc.); or 3) where the aspect for the particular site changed. Size and shape of the talus patch determined the number and length of transects [40]. There were 1–5 transects per site that ranged in length from 53 to 254 m. In cases where sites were established within an entire hillside or ridgeline of continuous talus habitat, we sampled three transects 210 m in length. While the size of pika territories can vary, we assumed them to average ~30 m in diameter [12]. Our approach, therefore, allowed for survey of ~2–9 possible pika territories per transect but ultimately accounted for scat within 2 m of the transect line to give us an estimate of scat density. This variable sampling effort, dependent on site size, was similar to that employed by another recent paper on pika distribution [22]. We decreased the 210 m standard to two 150 m transects for the Bighorn sites in 2011 to increase survey efficiency. We considered this reasonable because we maintained consistent transect placement and sampling methods. Parallel transects were 60 m from one another on the slope except for sites smaller than 8 pika home ranges. In such cases, we placed parallel transects 30 m from one another to allow for approximately 1 pika home range between surveyors (6 sites of 83).

Surveyors slowly moved across talus slopes perpendicular to the fall line searching along a given transect for all scat within 2 m on either side, including within crevices and rock interstices. We counted pellets singly up to piles of 25, and due to the difficulty of reliably distinguishing between old and new scat, did not record these separately. If a pile had more than 25 pellets, we simply recorded it as 25. Another study has used this number as a threshold to determine site occupancy [18]. Pika scat tends to be clumped, which made it suitable to record pellets within 1 m of a pile as part of the same pile. We calculated density of scat by dividing by the number of meters surveyed for all transects at a given site. In a subset of resampled sites, scat counts were significantly higher in 2011 (2010: *X* = 0.28, *SD* = 0.20; 2011: *X* = 0.57, *SD* = 0.31; t(9) = -6.015, *P* < 0.001). However, scat counts by site across years were highly correlated (*r* = 0.93, N = 7, *P* < 0.001).

### Surveyor Bias

Four observers participated in our surveys in each year (2010 and 2011), with two participating both years, for a total of six unique observers. To minimize potential observer bias, each year observers were randomly assigned across varying site strata including elevation, aspect and slope. To test for potential bias, in 2011 each of the four researchers surveyed the same extra 60-m “test transects” (n = 10). We used these data in a one-way ANOVA to test for observer differences. There was no difference between observers (*F*_2, 27_ = 0.69, *P* > 0.05), and we assumed potential observer differences were similar in 2010.

### Forage Availability

We measured forage availability via two metrics: the abundance of foraging habitat within a patch (“patch forage”) and the proportion of the perimeter of the talus patch that was foraging habitat (“perimeter forage”). At each site we surveyed forage availability from the middle of the second transect or from a haypile within 12 m of that point when available and used a modified point-intercept method [16]. In a few cases, the middle of the second transect was considered unrepresentative of the site because it landed either on a rock outcropping or in the center of the only meadow in the site. In such cases, we moved the survey point down slope 30 m. At each survey point for patch forage, we established two perpendicular 50-m transects along which vegetation cover types were recorded at 1-m intervals (n = 100 points per survey). The number of points out of 100 that touched potential forage was our estimate of percent forage availability. We included grasses, forbs, shrubs, trees, cushion plants, mosses, and ground lichens as available forage because 1) pikas are generalist foragers [41] and 2) we observed all of these forage types in pika haypiles.

We also estimated forage availability around the edge of each site to account for sites that are primarily rock within the talus patch but have potential foraging areas around the perimeter [42]. We designated each meter along the scat survey line as available forage if there was a distinct edge of meadow within 15 m (visual estimate of greater than 75% vegetation cover). We divided the number of meters characterized as available forage by the total meters surveyed to calculate the proportion of perimeter forage availability for each site.

### Climatic Variables

We used Thermochron iButton temperature sensors to measure ambient temperatures at sites in the Winds during 19 September 2010 through 17 August 2011 (model DS1921G; temperature ranges -40°C to 85°C). We set all sensors to track temperature every four hours (02:00, 06:00, 10:00, 14:00, 18:00, and 22:00 hours each day). We deployed a sensor at the approximate center of each survey site, near a haypile when available, to record ambient temperatures that a pika at that site would likely experience. We sealed each sensor in a 0.5-ounce clear plastic case with approximately one half of a teaspoon of anhydrous calcium chloride desiccant (DampRid). Each sensor was affixed to a rock using clear polyvinyl tape and heavy-duty weed whacker line. The sensor was deployed approximately 0.75 m below the talus surface. Upon retrieval, several loggers were either missing, moved, or exposed to direct sunlight. Out of 43 sites, we retrieved 27 subsurface loggers that tracked temperature and were not exposed to direct sunlight.

We derived nine climate variables from the temperature sensor data related to our three hypotheses: number of days above 15°C, number of days temperature reaching <0, <-5, and <-10°C, mean winter temperature; number of days above 10°C, total degree days, length of growing season, and mean summer temperature (Table 1). Our sampled temperature values were substantially lower than the previously identified limiting upper temperature threshold for pikas of 26°C [12]. Heat stress is therefore likely not a strong limiting factor at our sites. Temperatures that elicit heat stress, however, may vary geographically due to local adaptation. We therefore used the number of days above 15°C, which represented the upper 99th percentile of temperatures documented at our sites, to examine potential effects of higher relative temperatures on pikas. We counted the number of days where any temperature value was below 0°C as a measure of length of winter, and number of days below -5 and -10°C to test for two proposed thresholds for acute cold stress on pikas [11, 16]. We used all days with any temperature value above 0°C as the length of the potential growing season and calculated the mean temperature of those days as an estimate of mean summer temperature. We also calculated average total degree days in each growing season by multiplying the length of the growing season by the mean temperature during the growing season. The mean summer temperature, total degree days, and length of growing season variables were metrics of climate conditions suitable for vegetation growth. A robust body of literature (e.g., [43–44]) has documented increased plant growth with higher temperatures at high elevations and latitudes. Length of the growing season is indicative of the absence of snow, onset of timing of green-up and overall biomass in addition to other factors [45]. Because our mean summer temperature values were substantially lower in comparison to established lethal thresholds for pikas from previous studies [12, 23], we conjectured that its dominant influence on pikas would be through its effects on plant growth, and hence food production. We therefore used mean summer temperature to test our forage availability rather than summer heat hypothesis. Total food available for pikas should be the product of vegetated area (our measures of forage from the field) and productivity, indexed by these weather variables. These two sets of factors could both contribute to pika abundance, as, unsurprisingly, they were not strongly related to one another (Table D in S1 File).

We also tested several other climatic variables derived from interpolated and remotely sensed precipitation (PRISM), temperature (PRISM), and snow cover data (MODIS and SnoDAS). These data sources are often used in wildlife studies to quantify climate [46–48], however, in our study, none of these had significant predictive power, likely due to a mismatch in relevant spatial scales. Accordingly, we do not discuss these remotely sensed or interpolated climate measures any further.

### Other Site Characteristics

Talus interstices provide pikas refugia from predators and facilitate body temperature regulation. We estimated talus depth as an indicator of interstices at every 30 m along each transect in 2010 and every 3 m in 2011. A 2-m snow probe was laid on the surface of the talus to approximate the surface of a 1-m radius plot. Within the 1-m radius plot, we measured the vertical distance between the bottom of the deepest crevice to the talus surface to the nearest 0.5 m. Finally, we refined field estimations of elevation and aspect and quantified elevation difference to summit using ArcGIS version 9.3. We estimated the elevation difference to the summit to capture any effect of exposure to wind and therefore exposure to extreme temperatures during winter and desiccation, which may vary across a mountain range [49].

### Statistical Analyses

We tested the support for our three hypotheses (*summer heat*, *winter cold*, *and forage availability*) using an information theoretic approach. We used habitat and climatic variables in three different tests to predict relative pika abundance, as indexed by scat density. First, we tested the predictive power of habitat characteristics to explain scat densities by developing 39 general linear models (GLMs) that included different combinations of linear and quadratic effects of 6 habitat variables (See Tables A and B in S1 File for a full list of models). These explanatory variables were elevation, aspect, and elevation difference to summit as potential proxies of climate, and talus depth and the two metrics of forage availability to account for habitat features expected to be meaningful to pikas though not necessarily indicators of climate. We used Akaike’s Information Criteria corrected for small sample size (AIC_*c*_) [50] to select the best-supported models for both datasets (Winds 2010 and Bighorns 2011). The AIC_*c*_ framework allowed us to identify patterns observed in each mountain range separately to evaluate the generality of the top models and avoid the need to test extremely complex models that included multiple interactions of mountain range with other factors.

Second, using the best-supported model from the first analysis, we replaced the linear and squared terms of elevation with each of the nine temperature sensor variables derived from the ibutton data at the 27 Winds sites. This suite of models was composed of ten candidate models, including the original elevation model. We again used AIC_*c*_ to determine which climate factors best-predicted scat density.

Finally, we evaluated a set of 220 candidate general linear models that included combinations of linear and squared terms of relatively uncorrelated (r < 0.5) temperature sensor variables, with patch forage (See Table C in S1 File for a full list of models). The aim here was to determine if there was a better model for predicting scat density than one based on the original elevation model, and using a broader range of combinations of climate measures.

## Results

### Habitat Features Model Selection

#### Wind River Range

**Fig 1:**
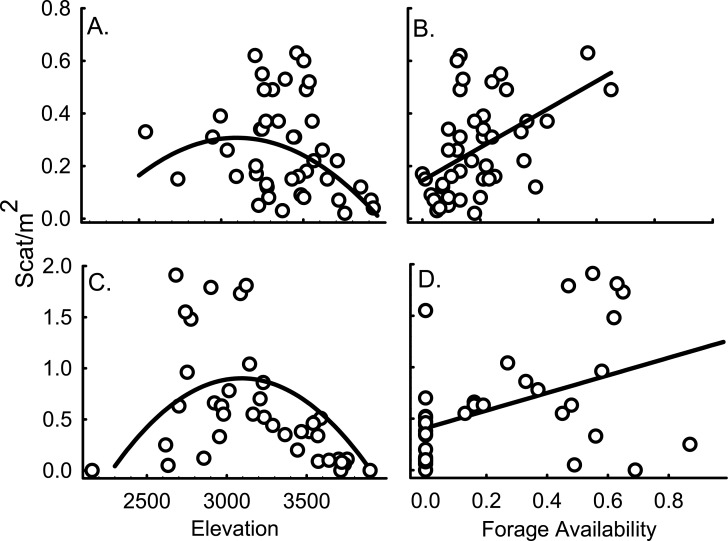
American pika scat density as a function of top habitat predictor variables. Scat/m^2^ as a function of elevation (a) and patch forage availability (b) in the Wind River Range, Wyoming, USA, 2010 (adj. r^2^ = 0.32), and elevation (c) and perimeter forage availability (d) in the Bighorn Range in 2011 (adj. r^2^ = 0.40). Lines in each panel represent the predicted relationship of the top model for each range, holding patch forage (a), perimeter forage (c), and elevation (b, d) at their mean values.

American pika scat/m^2^ ranged from 0.04 to 0.63 at sites (n = 43) in the Winds in 2010 with a mean of 0.27 ± 0.027 SE. The AIC_*c*_ model with the strongest support included linear and quadratic terms of elevation and a linear term of patch forage, with other well-supported models including similar variables (Table 2). A null model had very little support (AIC_*c*_ = -22.8; ΔAIC_*c*_ = 12.6; Akaike Weight = 0.00). Summed Akaike weights of all models that included elevation and patch forage showed strong support for those variables (summed Akaike weights = 0.73 and 0.98, respectively). Scat density increased with elevation to an approximate apex at about 3600 m, beyond which density decreased (Fig 1A). The best-supported relationship between patch forage and scat/m^2^ was linear and positive (Fig 1B). Other variables had little to no support as predictors of scat density, including aspect, talus depth, and elevation difference to summit.

**Table 2:** Results of general linear models of American pika scat density in the Wind River and Bighorn Ranges, Wyoming, USA (2010–2011) in relation to habitat variables with models displayed based on Akaike’s information criterion corrected for small sample size (AIC_*c*_).

Top models	*n*	*k*	AIC_*c*_	ΔAIC_*c*_	*w*_*i*_	adj r^2^
**Winds**						
(elevation), (elevation^2^), (patch forage)	43	5	-35.39	0	0.44	0.32
(elevation), (elevation^2^), (patch forage), (patch forage^2^)	43	6	-33.02	2.36	0.14	0.31
(elevation), (patch forage)	43	4	-32.54	2.85	0.11	0.25
(patch forage), (difference to summit)	43	4	-31.64	3.74	0.07	0.24
Null model	43	2	-22.71	12.6	0.00	0.00
**Bighorns**						
(elevation), (elevation^2^), (perimeter forage)	40	5	52.05	0.0	0.38	0.41
(elevation), (elevation^2^), (perimeter forage), (perimeter forage^2^)	40	6	54.10	2.05	0.14	0.40
(elevation), (elevation^2^), (patch forage)	40	5	54.69	2.65	0.10	0.37
(elevation), (elevation^2^)	40	4	54.79	2.74	0.09	0.34
(elevation), (elevation^2^), (aspect)	40	5	55.81	3.76	0.06	0.35
Null model	40	2	68.69	16.6	0.00	0.00

Models within 4 ΔAIC_***c***_ units of the top model are shown. Model weights (*w*_*i*_) were calculated for each model based on the number of parameters (*k*) and number of sites sampled (*n*). See Tables A and B in S1 File for full model sets.

#### Bighorn Range

Scat/m^2^ ranged from < 0.01 to 1.91 at the Bighorns sites (n = 40) with a mean of 0.59 ± 0.087 SE. Patterns of scat density were strikingly similar to those in the Winds. Scat density was highest at mid elevations and increased linearly with perimeter forage (Fig 1C and 1D). The best-supported model included linear and quadratic terms of elevation and a linear term of perimeter forage, followed by models with similar terms (Table 2). The top model had substantial support in comparison to most other models, including the null (AIC_*c*_ = 63.1; ΔAIC_*c*_ = 11.1; Akaike Weight = 0.00). Though elevation and perimeter forage were correlated (r = -0.74; see Table D in S1 File for correlation values of habitat variables from both ranges), there was substantial support for inclusion of both factors in explaining pika scat densities. The difference between the AIC_*c*_ value for the best model containing elevation but not forage perimeter, and the top model which included both effects, was 2.08, indicating the additive contribution of both factors [50]. Summed Akaike weights of all models that included elevation and perimeter forage showed strong and moderate support for those variables, respectively (summed Akaike weights: elevation = 0.95; perimeter forage = 0.54). Scat density as a function of elevation decreased sharply at approximately 3300 m (Fig 1C).

**Fig 2:**
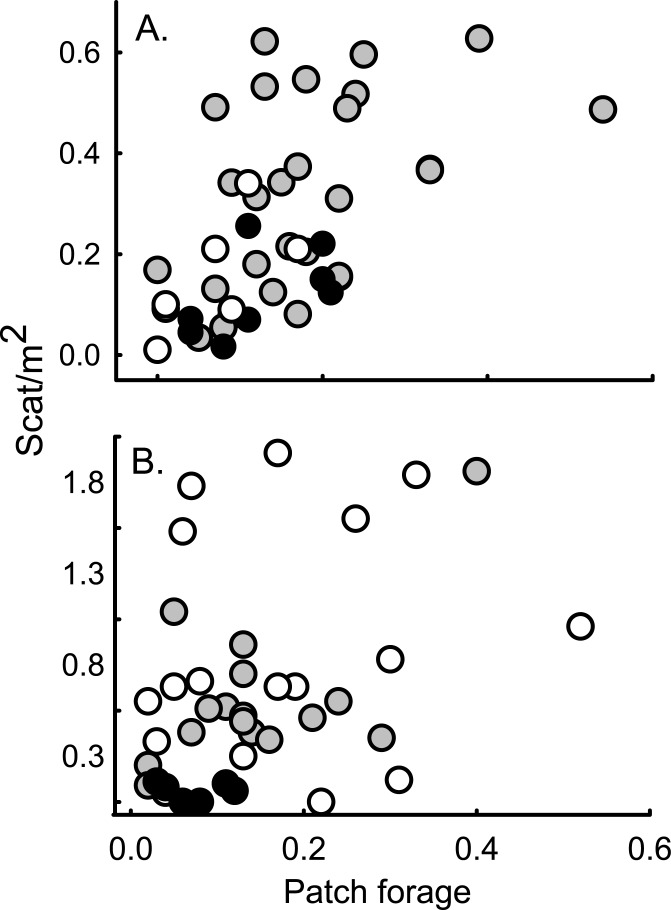
American pika scat density as a function of forage availability across elevation. Scat/m^2^ in the Wind River (a; n = 43 sites) and Bighorn (b; n = 40) ranges in Wyoming, USA, 2010 and 2011, respectively. Elevation ranges are: < 3100m (white), > 3100 and < 3600m (gray), > 3600m (black); n = 43 sites.

A narrow elevation band (~600 m) specific to each mountain range (Winds: ~3000–3600 m and Bighorns: ~2700–3300 m) contained the widest range of scat density (Fig 1A and 1C). This elevation band was shifted about 300 m higher in the Winds, which could be an effect of difference in latitude between the two ranges. The shape of the observed pattern, however, held for both mountain ranges. Both patch forage and scat density were consistently low at high elevations (Fig 2). For further details on the data, see Tables A and B in S2 File.

### Climate Data Model Selection

**Fig 3:**
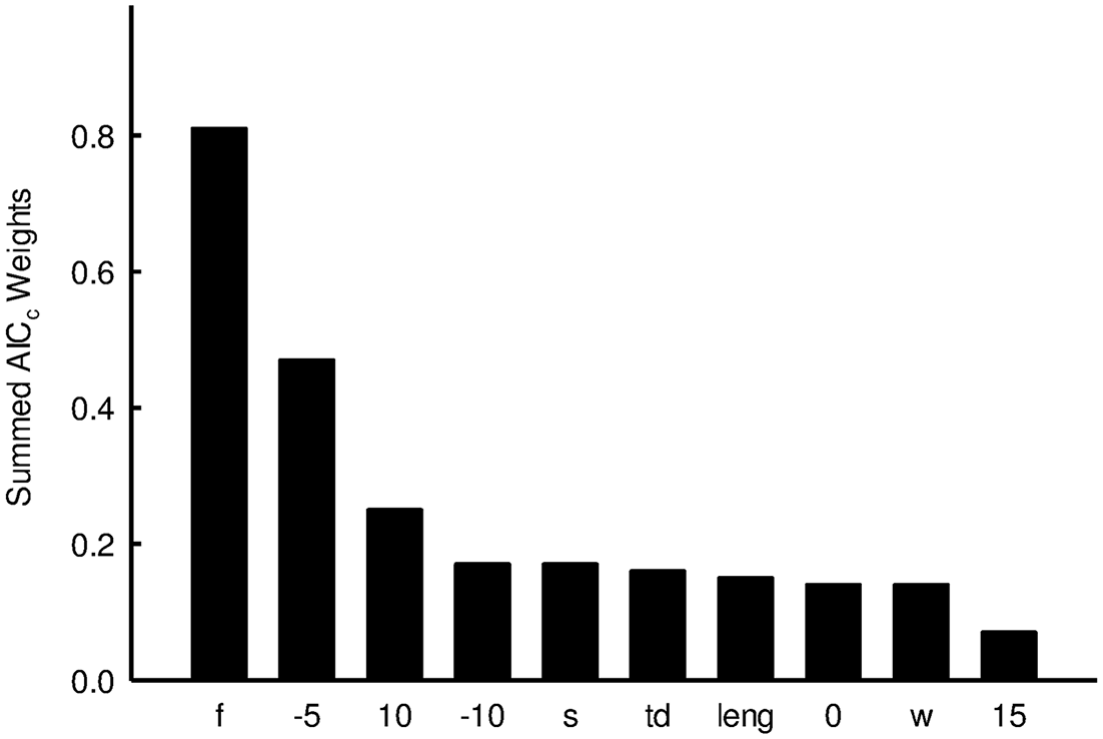
Summed Akaike weights for local microclimate and forage predictor variables. Summed Akaike weights indicating overall support for predictor variables (f = forage; -5 = number of days below -5°C; 10 = number of days above 10°C; -10 = number of days below -10°C; s = mean summer temperature; td = total degree days; leng = length of the growing season; 0 = days below 0°C; w = mean winter temperature; 15 = number of days above 15°C) in relation to American pika scat density in Wyoming, USA across all models tested.

AIC_*c*_ results from the suite of models that substituted different local climate variables for elevation largely agreed with the analyses of a larger suite of models (Tables 3 and 4). In particular, patch forage was present in most well-supported models in each suite, and two climate variables—days below -5°C and days above 10°C—were also present in virtually all well-supported models (Tables 3 and 4). Other climate variables that were present in well-supported models included mean summer temperature, mean winter temperature, total degree days, days below -10°C, and growing season length. However, the summed AIC_*c*_ weights, which indicate across-model support for a variable, where by far the highest for the top three variables: forage, days below -5°C, and days above 10°C (Fig 3).

**Table 3:** Results of models fit to relative American pika scat density at a subset (n = 27) of Wind River Range (Wyoming, USA) sites (2010) using temperature sensor data showing the five best-supported models that replaced elevation with climate effects, as well as the best habitat variable model and the null model.

Top models	*n*	*k*	AIC_c_	ΔAIC_c_	*w*_*i*_	adj r^2^
(below -5), *(below-5)* ^*2*^, (forage)	27	5	-13.53	0.00	0.43	0.29
(above 10), *(above 10)* ^*2*^, (forage)	27	5	-11.40	2.13	0.15	0.24
(summer mean), *(summer mean)* ^*2*^, (forage)	27	5	-10.37	3.17	0.09	0.21
(total degree days), *(total degree days)* ^*2*^, (forage)	27	5	-9.55	3.98	0.06	0.18
*(winter mean)*, *(winter mean)* ^*2*^, (forage)	27	5	-9.28	4.25	0.05	0.17
Null model	27	2	-9.19	4.34	0.04	0.00
(elevation), *(elevation)* ^*2*^, (forage)	27	5	-8.87	4.66	0.04	0.16

Models were based on Akaike’s Information Criterion corrected for small sample size (AIC_*c*_). *Italicized* variables have negative coefficients; model weights (*w*_*i*_) were calculated for each model based on the number of parameters (*k*) and number of sites sampled (*n*).

**Table 4:** Results of models fit to relative American pika scat density at a subset (n = 27) of Wind River Range (Wyoming, USA) sites (2010) using iButton temperature sensor data showing the best models of the full suite.

Top models	*n*	*k*	AIC_*c*_	ΔAIC_*c*_	*w*_*i*_	adj r^2^
(above 10), (forage)	27	4	-14.38	0.00	0.05	0.27
(below -5), *(below -5)* ^*2*^	27	4	-13.86	0.52	0.04	0.25
(below -5), *(below -5)* ^*2*^, (forage)	27	5	-13.53	0.84	0.03	0.29
*(below -10)*, (above 10), (forage)	27	5	-13.49	0.89	0.03	0.29
(summer mean), (forage)	27	4	-13.29	1.09	0.03	0.24
(below -5), *(below -5)* ^*2*^, (total degree days), (forage)	27	6	-13.24	1.14	0.03	0.34
(below -5), (above 10), (forage)	27	5	-12.92	1.45	0.03	0.28
(below -5), *(below -5)* ^*2*^, (growing length)	27	5	-12.76	1.61	0.02	0.27
(below -5), (total degree days), (forage)	27	5	-12.68	1.70	0.02	0.27
(below -10), *(below -10)* ^*2*^, (above 10), (forage)	27	6	-12.59	1.78	0.02	0.32
(total degree days), (forage)	27	4	-12.59	1.79	0.02	0.22
(below -5), *(below -5)* ^*2*^, *(below 0)*	27	5	-12.53	1.85	0.02	0.27
(below -5), *(below -5)* ^*2*^, (growing length), (forage)	27	6	-12.49	1.88	0.02	0.32
Null model	27	2	-9.19	5.18	0.00	0.00

Models were based on Akaike’s Information Criterion corrected for small sample size (AIC_*c*_) with ΔAIC_*c*_ and models within 2 ΔAIC_*c*_ units of the top model are shown. Variables with negative coefficients are *italicized*. See Table C in S1 File for the full model suite.

**Fig 4:**
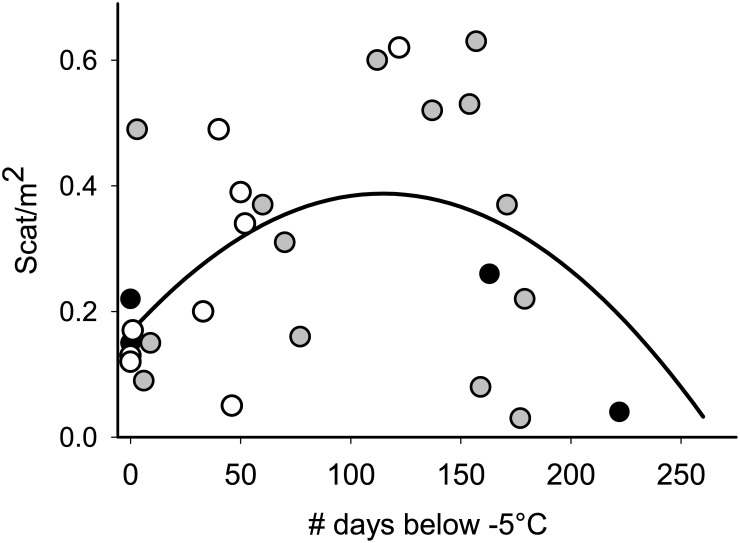
Relationship between American pika scat density and number of days below -5°C. Data were recorded with temperature loggers at 27 sites in the Wind River Range, Wyoming, USA from one year starting in August 2010. Colors represent different elevation ranges (white: < 3300m, (n = 9), gray: 3300-3600m, (n = 14); black: > 3600 m, (n = 4). The curve represents the predicted relationship from the top model of the temperature sensor analysis (adj. r^2^ = 0.29).

The climate metric with the highest support was days below -5°C, which always entered models with support for both linear and quadratic terms (Tables 3 and 4). Scat density showed a unimodal response to this variable, with the lowest scat/m^2^ at both the lowest and highest number of days below -5°C and the highest scat/m^2^ around the mid-range of days below -5°C (Fig 4). Notably, days below -5°C varies with elevation, with more colder days at higher elevation (r^2^ = 0.25; Fig 4).

**Fig 5:**
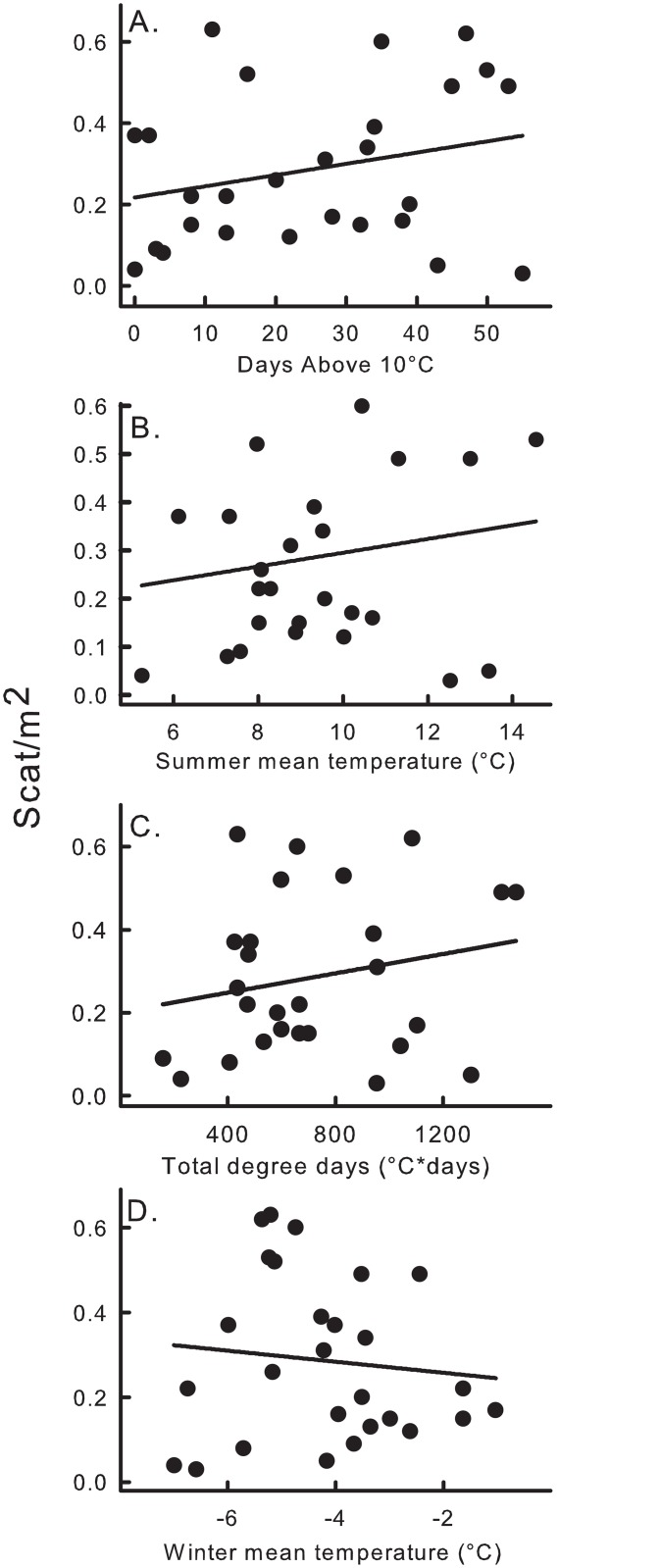
Correlations between four local climate measures and elevation. Climate variables included from top to bottom: number of days above 10°C, adj. r^2^ = 0.34; mean summer temperature, adj. r^2^ = 0.28; total degree days (°C*days), adj. r^2^ = 0.33; and mean winter temperature, adj. r^2^ = 0.09. All data were obtained via ibutton sensors deployed at 27 sites in the Wind River Range, Wyoming, USA for one year starting in August 2010.

**Fig 6:**
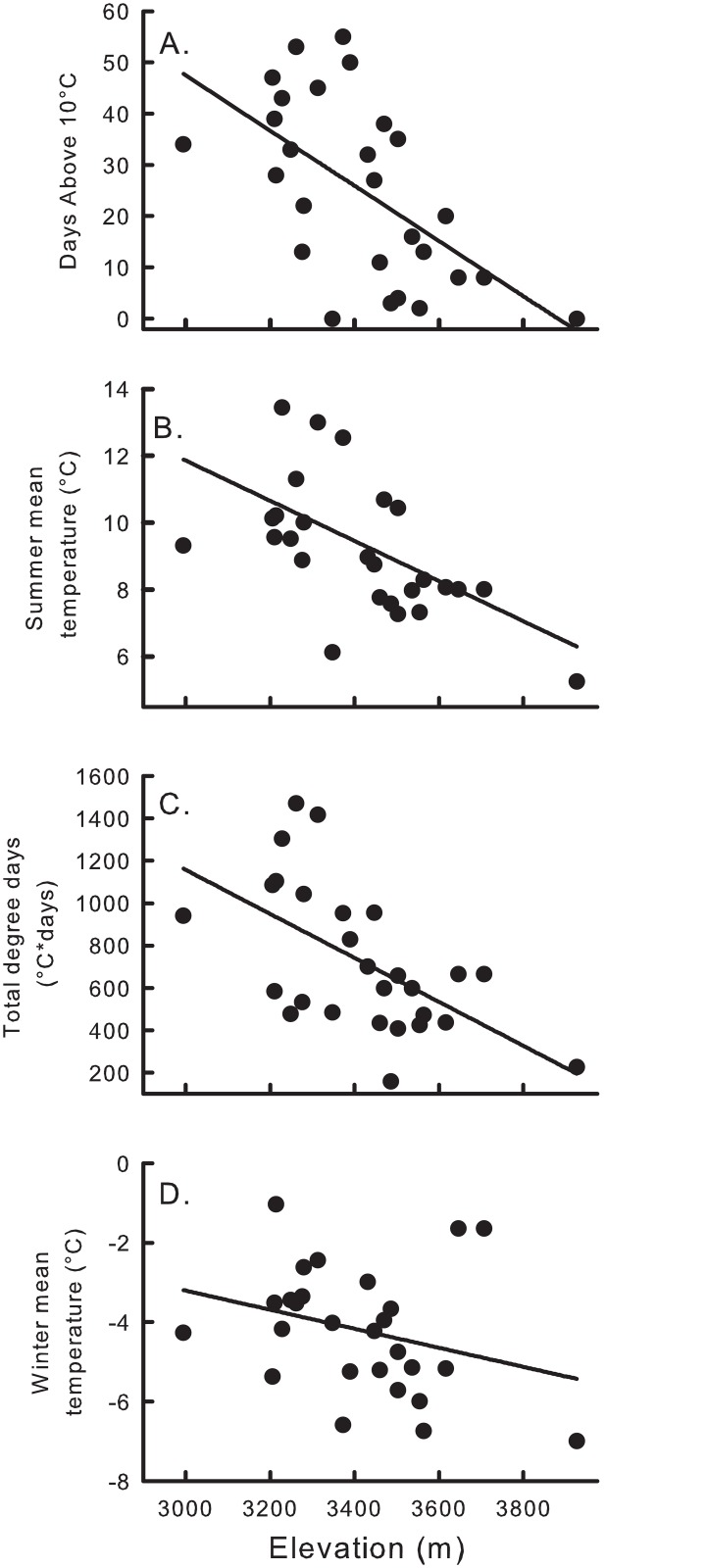
Correlations between American pika scat density and four local climate measures. Climate variables from top to bottom: number of days above 10°C, adj. r^2^ = 0.06; mean summer temperature, adj. r^2^ = 0.02; total degree days, adj. r^2^ = 0.04; and mean winter temperature, adj. r^2^ = 0.01; were obtained via ibutton sensors in the Wind River Range, Wyoming, USA deployed during August, 2010 –August 2011.

The other climate variables supported in one or the other model suite also varied with elevation (Fig 5). Days above 10°C, mean summer temperature, total degree days, and mean winter temperature, all included in models that outcompeted elevation in the original model, each declined with increasing elevation. Scat increased with mean summer temperature and total degree days, decreased with mean winter temperature, and showed a either unimodal relationship or linear increase with days above 10°C, depending on the model suite (Fig 6; Tables 3 and 4).

A model with only forage explained relatively little variance (adj. r^2^ = 0.16) compared to that of the best-supported model in the final model suite (adj. r^2^ = 0.34). There was limited support for days below 0°C, summer mean temperature, and total degree days which were inconsistently among some of the top models and had similar summed weights. The other variables we tested, including growing season length, winter mean temperature, days below -10°C, and days above 15°C, had much weaker explanatory power than did the best climate variables (Fig 3). For further details on the data, see S3 File.

## Discussion

Elevation is frequently used as a surrogate for climate in predicting species distributions. This is especially the case for shifts in range limits because temperature and sometimes other aspects of climate (i.e., precipitation), are strongly related to elevation. Many other important characteristics of habitat, however, can also co-vary with elevation, sometimes in complex ways. In the case of American pikas, forage availability is strongly associated with elevation, and therefore climate, mostly likely due to the relationship of environmental and climatic conditions across an elevation gradient (Figs 1 and 4), thereby complicating the interpretation of elevation as a simple proxy for abiotic conditions. Furthermore, both elevation and forage availability were strong predictors of scat density, with both factors independently influencing scat density. This relationship suggests that elevation indexes key climatic metrics, which appear to independently influence both forage availability and scat density. The similar patterns and explanatory support for elevation and forage availability in the two mountain ranges of study strengthen our inference that these are the primary limiting factors in this North-Central Rocky Mountain region, although these may differ from limitations in other parts of American pika range.

Our results supported the forage availability hypothesis in two ways. First, the best-supported model included a positive effect of forage availability on pika scat density. Forage availability within talus patches and around patch perimeters were among the best predictive variables for the Wind River and Bighorn ranges, respectively. While these metrics indexed different aspects of food availability, both are indicative of the same general effect. Since food availability commonly influences species’ abundance, the positive linear relationship of forage and scat density was not surprising. However, along with elevation, our results suggest that forage availability plays a particularly important role in this system.

Some alpine areas are comprised of talus/meadow mosaics and provide a heterogeneous landscape with strong microclimatic variation, which promotes species richness of plants, insects, and mammals [51–53]. Although pikas can range up to hundreds of meters in search of forage [54], such diverse and patchy landscapes allow pikas to collect hay more locally, which is more energetically efficient. Abundant and nearby forage enables pikas to cache as much vegetation as possible during the short alpine growing season while still being able to defend collected hay. Pikas may also consume uncollected forage within their territory if accessible under the snow during winter, which can improve the odds of over-winter survival. Another typical kind of talus patch consists of large lobes of pure talus with very little forage available within the talus matrix. Often, these patches are characterized by a distinct talus-meadow edge and high density of pikas near the interface, which provides an abundant and diverse food source [12, 42]. Such nearby meadows are often local hot spots for biodiversity [55] and may allow pikas to selectively collect forage for both summer and winter diets [56]. Forage availability in both types of suitable talus patches, one with a distinct talus-meadow interface and the other with patchy meadows among the talus field, was an important predictor of scat density.

The forage availability hypothesis was also supported by the climatic variables we tested. Climate factors indicating warmer summer conditions (mean summer temperature, days above 10°C, and total degree days) had substantial explanatory strength. All of these factors were positively associated with scat density, and these relationships are likely related to food availability. Several experimental manipulations have shown that environmental conditions such as earlier springs and warmer growing season temperatures allow for increased plant growth and reproduction in high elevation ecosystems [44,57,58]. For herbivores, plant productivity enhances body condition and potential fitness, which may scale up to influence abundance and distributions [59]. Favorable climatic conditions can also promote higher quality forage for pikas [22]. Pika scat density was higher at sites where climate factors were favorable for forage growth suggesting that limitation on pika distributions at this latitude are dependent on plant growing conditions. Temperature sensors were placed within the talus, so caution is needed in interpreting the recorded temperatures as those experienced by forage plants. We assumed, however, that these temperatures were correlated with those experienced by surrounding vegetation within the study site.

Our results also supported predictions of the winter snowpack hypothesis. The number of days below -5°C was positively correlated with scat density up to about 120 days, and negatively related at higher values. Because the days below -5°C variable indicates where temperatures are low and thermal insulation is weak, the result emphasizes the importance of insulative snowpack for pikas, but only at relatively high numbers of cold days. Across all elevations, several sites showed lower scat density with low numbers of particularly cold days. This result suggests that up to a point, more cold days may be beneficial to pikas, perhaps because they indicate conditions in which a stable insulating snowpack can be established. The relationship may reach a threshold, however, beyond which there are simply too many very cold days for pikas to withstand, such as on high elevation, wind-scoured slopes.

Our work highlights the context-dependent nature of climatic effects on species across elevations within and across regions. The factors limiting American pikas across the two mountain ranges in the North-Central Rocky Mountains in our study appear to be different than correlates limiting pika range limits in other regions such as the Great Basin [11, 15, 16], where combinations of acute cold, and heat in addition to other factors appear to be important drivers of persistence. We emphasize that the highest elevation sites may not be a suitable refuge from climate change at all latitudes. Pikas may already be at their upper elevation limit in parts of their range [60], which is contrary to the idea that conditions at lower elevations are the primary limit on current pika distributions. With a lack of extreme high values in our temperature data and little support for the above 15°C climate variable, there was no support for the summer heat hypothesis, suggesting that pikas at this latitude are currently not limited by hot summer temperatures. Elevation may limit pikas and other alpine species in future warmer years, but we expect that these physiological limits will be strongly modified by constraints imposed by food availability. Our results demonstrate a complex set of climatic and elevation effects that make forecasting from current elevation and climate relationships to future conditions more ambiguous. In future warmer years, additive and interactive effects of climatic conditions and alpine habitat distribution will likely influence pika range shifts. Further work investigating the influence of climatic variables on abundance (e.g., [17]) as well as specific forage requirements would add to our understanding of the complexities of this climate-species relationship, and facilitate the predictability of pika and alpine-meadow distribution and abundance.

While several alpine species worldwide have responded to changing climate by moving upslope [8, 61, 62], our findings suggest that such responses by American pikas could be severely limited by the interactive effects of elevation and food availability and quality. Under current conditions there appears to be broad overlap of suitable vegetation communities and climate conditions for pikas in the North-Central Rocky Mountains, but these zones might diverge with rapid climate change. Plant species within the alpine zone are likely to respond in a variety of ways to climate change, but in general, development of highly productive alpine meadows at higher elevations will require substantial soil development, resulting in long lags between warming temperatures and establishment of suitable forage conditions for pikas [63, 64]. We speculate that a combination of rapid upward movement of climate conditions suitable for pikas, but only slow migration of suitable plant communities, is likely to create a much narrower zone of inhabitable conditions for pikas over the near term. There is ongoing conservation concern for this species that resulted in a petition for listing under the Endangered Species Act in 2010. As climate change continues and novel climatic conditions continue to emerge, implications for the American pika will likely involve combinations of physiological and/or food resource limitation. We suggest that effective pika conservation will therefore require a comprehensive and multifaceted consideration of this species’ limitations.

## Supporting Information

S1 File Supporting information for model results and correlation matrices. General linear model and AIC_*c*_ model selection results—Wind River Range 2010 (**Table A**). General linear model and AIC_*c*_ model selection results—Bighorn Range 2011 (**Table B)**. General linear model selection AIC_*c*_ results—subset of Wind River sites 2010 (**Table C)**. Correlation matrix for habitat variables in the Wind River Range 2010 and Bighorn Range 2011 (**Table D**). Correlation matrix for forage variables and climate variables in the Wind River Range 2010 (**Table E**).
(DOCX)

S2 File All habitat and scat data for each site collected in 2010 for the Wind River Range, WY (n = 43; Table A) and in 2011 for the Bighorn Range (n = 40; Table B). (DOCX)

S3 File Climate data derived from temperature-sensors data collected 19 September 2010 through 17 August 2011 for sites in the Wind River Range (n = 27). (DOCX)
