# Supplementary material for: Climate Tolerances and Habitat Requirements Jointly Shape the Elevational Distribution of the American Pika (Ochotona princeps), with Implications for Climate Change Effects
Source: PLoS One. 2015 Aug 5;10(8):e0131082. doi: 10.1371/journal.pone.0131082 (PMC4526653; doi:10.1371/journal.pone.0131082)
Supplement: S1 File — General linear model and AICc model selection results—Wind River Range 2010 (Table A). General linear model and AICc model selection results—Bighorn Range 2011 (Table B). General linear model selection AICc results—subset of Wind River sites 2010 (Table C). Correlation matrix for habitat variables in the Wind River Range 2010 and Bighorn Range 2011 (Table D). Correlation matrix for forage variables and climate variables in the Wind River Range 2010 (Table E). (DOCX) [file pone.0131082.s001.docx]

**S1 File.**

**Table A. General linear model and AIC*_c_* model selection results for the Wind River Mountains sampled 2010.**

| *model* | *elevation* | *elevation^2^* | *patch forage* | *patch forage^2^* | *depth* | *depth^2^* | *aspect* | *difference to near summit* | *perimeter forage* | *perimeter forage^2^* | *n* | *k* | AIC*_c_* | ΔAIC*_c_* | *w_i_* | adj. r^2^ |
| --- | --- | --- | --- | --- | --- | --- | --- | --- | --- | --- | --- | --- | --- | --- | --- | --- |
| 22 | x | x | x |  |  |  |  |  |  |  | 43 | 5 | -35.39 | 0.00 | 0.45 | 0.32 |
| 23 | x | x | x | x |  |  |  |  |  |  | 43 | 6 | -33.03 | 2.36 | 0.14 | 0.31 |
| 20 | x |  | x |  |  |  |  |  |  |  | 43 | 4 | -32.54 | 2.85 | 0.11 | 0.25 |
| 38 |  |  | x |  |  |  |  | x |  |  | 43 | 4 | -31.64 | 3.75 | 0.07 | 0.24 |
| 18 |  |  | x |  |  |  |  |  |  |  | 43 | 3 | -30.78 | 4.61 | 0.04 | 0.20 |
| 21 | x |  | x | x |  |  |  |  |  |  | 43 | 5 | -30.22 | 5.17 | 0.03 | 0.24 |
| 39 |  |  | x | x |  |  |  | x |  |  | 43 | 5 | -29.74 | 5.64 | 0.03 | 0.23 |
| 30 |  |  | x |  |  |  |  |  | x |  | 43 | 4 | -29.29 | 6.10 | 0.02 | 0.20 |
| 24 |  |  | x |  | x |  |  |  |  |  | 43 | 4 | -29.13 | 6.26 | 0.02 | 0.19 |
| 28 |  |  | x |  |  |  | x |  |  |  | 43 | 4 | -29.10 | 6.29 | 0.02 | 0.19 |
| 31 |  |  | x |  |  |  |  |  | x | x | 43 | 5 | -28.83 | 6.56 | 0.02 | 0.21 |
| 19 |  |  | x | x |  |  |  |  |  |  | 43 | 4 | -28.58 | 6.81 | 0.01 | 0.18 |
| 40 |  |  |  |  |  |  |  |  |  |  | 43 | 2 | -22.76 | 12.62 | 0.00 |  |
| 32 |  |  | x | x |  |  |  |  | x |  | 43 | 5 | -26.99 | 8.39 | 0.01 | 0.18 |
| 29 |  |  | x | x |  |  | x |  |  |  | 43 | 5 | -26.98 | 8.41 | 0.01 | 0.18 |
| 27 |  |  | x | x | x |  |  |  |  |  | 43 | 5 | -26.87 | 8.51 | 0.01 | 0.18 |
| 25 |  |  | x |  | x | x |  |  | x |  | 43 | 5 | -26.63 | 8.76 | 0.01 | 0.17 |
| 33 |  |  | x | x |  |  |  |  | x | x | 43 | 6 | -26.43 | 8.96 | 0.01 | 0.20 |
| 5 | x | x |  |  | x |  |  |  |  |  | 43 | 5 | -25.33 | 10.05 | 0.00 | 0.14 |
| 26 |  |  | x | x | x | x |  |  |  |  | 43 | 6 | -24.23 | 11.15 | 0.00 | 0.16 |
| 2 | x | x |  |  |  |  |  |  |  |  | 43 | 4 | -24.00 | 11.38 | 0.00 | 0.09 |
| 3 | x |  |  |  | x |  |  |  |  |  | 43 | 4 | -23.44 | 11.95 | 0.00 | 0.08 |
| 1 | x |  |  |  |  |  |  |  |  |  | 43 | 3 | -22.76 | 12.62 | 0.00 | 0.03 |
| 6 | x | x |  |  | x | x |  |  |  |  | 43 | 6 | -22.62 | 12.76 | 0.00 | 0.12 |
| 7 |  |  |  |  |  |  | x |  |  |  | 43 | 3 | -22.37 | 13.01 | 0.00 | 0.02 |
| 11 | x | x |  |  |  |  | x |  |  |  | 43 | 5 | -22.24 | 13.15 | 0.00 | 0.08 |
| 12 | x |  |  |  | x |  | x |  |  |  | 43 | 5 | -21.88 | 13.50 | 0.00 | 0.07 |
| 10 | x |  |  |  |  |  | x |  |  |  | 43 | 4 | -21.81 | 13.57 | 0.00 | 0.04 |
| 36 | x | x |  |  |  |  |  |  | x |  | 43 | 5 | -21.43 | 13.95 | 0.00 | 0.06 |
| 17 | x | x |  |  | x |  |  | x |  |  | 43 | 5 | -21.43 | 13.95 | 0.00 | 0.06 |
| 8 |  |  |  |  | x |  | x |  |  |  | 43 | 4 | -21.43 | 13.96 | 0.00 | 0.03 |
| 4 | x |  |  |  | x | x |  |  |  |  | 43 | 5 | -20.87 | 14.52 | 0.00 | 0.05 |
| 15 |  |  |  |  |  |  | x | x |  |  | 43 | 4 | -20.71 | 14.68 | 0.00 | 0.02 |
| 34 | x |  |  |  |  |  |  |  | x |  | 43 | 4 | -20.35 | 15.03 | 0.00 | 0.01 |
| 16 | x |  |  |  |  |  |  | x |  |  | 43 | 4 | -20.33 | 15.06 | 0.00 | 0.01 |
| 37 | x | x |  |  |  |  |  |  | x | x | 43 | 6 | -20.22 | 15.17 | 0.00 | 0.07 |
| 14 | x | x |  |  | x | x | x |  | x | x | 43 | 7 | -20.14 | 15.25 | 0.00 | 0.12 |
| 35 | x |  |  |  |  |  |  |  | x | x | 43 | 5 | -19.39 | 15.99 | 0.00 | 0.02 |
| 13 | x |  |  |  | x | x | x |  |  |  | 43 | 6 | -19.17 | 16.21 | 0.00 | 0.05 |
| 9 |  |  |  |  | x | x | x |  |  |  | 43 | 5 | -18.91 | 16.48 | 0.00 | 0.01 |

**Table B. General linear model and AIC*_c_* model selection results for the Bighorn Mountains sampled 2011.**

| *model* | *elevation* | *elevation^2^* | *patch forage* | *patch forage^2^* | *depth* | *depth^2^* | *aspect* | *difference to near summit* | *perimeter forage* | *perimeter forage^2^* | *n* | *k* | AIC*_c_* | ΔAIC*_c_* | *w_i_* | adj. r^2^ |
| --- | --- | --- | --- | --- | --- | --- | --- | --- | --- | --- | --- | --- | --- | --- | --- | --- |
| 36 | x | x |  |  |  |  |  |  | x |  | 40 | 5 | 52.05 | 0.00 | 0.38 | 0.41 |
| 37 | x | x |  |  |  |  |  |  | x | x | 40 | 6 | 54.10 | 2.05 | 0.14 | 0.40 |
| 22 | x | x | x |  |  |  |  |  |  |  | 40 | 5 | 54.69 | 2.65 | 0.10 | 0.37 |
| 2 | x | x |  |  |  |  |  |  |  |  | 40 | 4 | 54.79 | 2.74 | 0.10 | 0.34 |
| 11 | x | x |  |  |  |  | x |  |  |  | 40 | 5 | 55.81 | 3.76 | 0.06 | 0.35 |
| 6 | x | x |  |  | x | x |  |  |  |  | 40 | 6 | 56.67 | 4.62 | 0.04 | 0.36 |
| 5 | x | x |  |  | x |  |  |  |  |  | 40 | 5 | 56.73 | 4.68 | 0.04 | 0.33 |
| 17 | x | x |  |  |  |  |  | x |  |  | 40 | 5 | 57.00 | 4.95 | 0.03 | 0.33 |
| 23 | x | x | x | x |  |  |  |  |  |  | 40 | 6 | 57.47 | 5.43 | 0.03 | 0.35 |
| 31 |  |  | x |  |  |  |  |  | x | x | 40 | 5 | 57.75 | 5.70 | 0.02 | 0.32 |
| 14 | x | x |  |  | x | x | x |  |  |  | 40 | 7 | 57.94 | 5.89 | 0.02 | 0.37 |
| 13 | x |  |  |  | x | x | x |  |  |  | 40 | 6 | 59.47 | 7.42 | 0.01 | 0.32 |
| 30 |  |  | x |  |  |  |  |  | x |  | 40 | 4 | 59.49 | 7.44 | 0.01 | 0.26 |
| 4 | x |  |  |  | x | x |  |  |  |  | 40 | 5 | 59.55 | 7.50 | 0.01 | 0.28 |
| 33 |  |  | x | x |  |  |  |  | x | x | 40 | 6 | 60.19 | 8.14 | 0.01 | 0.30 |
| 35 | x |  |  |  |  |  |  |  | x | x | 40 | 5 | 61.07 | 9.02 | 0.00 | 0.26 |
| 12 | x |  |  |  | x |  | x |  |  |  | 40 | 5 | 61.23 | 9.18 | 0.00 | 0.25 |
| 32 |  |  | x | x |  |  |  |  | x |  | 40 | 5 | 61.96 | 9.91 | 0.00 | 0.24 |
| 3 | x |  |  |  | x |  |  |  |  |  | 40 | 4 | 62.01 | 9.96 | 0.00 | 0.21 |
| 20 | x |  | x |  |  |  |  |  |  |  | 40 | 4 | 62.33 | 10.28 | 0.00 | 0.20 |
| 10 | x |  |  |  |  |  | x |  |  |  | 40 | 4 | 62.38 | 10.34 | 0.00 | 0.20 |
| 34 | x |  |  |  |  |  |  |  | x |  | 40 | 4 | 62.60 | 10.55 | 0.00 | 0.20 |
| 1 | x |  |  |  |  |  |  |  |  |  | 40 | 3 | 63.14 | 11.09 | 0.00 | 0.16 |
| 28 |  |  | x |  |  |  | x |  |  |  | 40 | 4 | 63.82 | 11.77 | 0.00 | 0.17 |
| 18 |  |  | x |  |  |  |  |  |  |  | 40 | 3 | 64.36 | 12.31 | 0.00 | 0.13 |
| 16 | x |  |  |  |  |  |  | x |  |  | 40 | 4 | 64.85 | 12.80 | 0.00 | 0.15 |
| 38 |  |  | x |  |  |  |  | x |  |  | 40 | 4 | 64.91 | 12.86 | 0.00 | 0.15 |
| 21 | x |  | x | x |  |  |  |  |  |  | 40 | 5 | 64.95 | 12.90 | 0.00 | 0.18 |
| 24 |  |  | x |  | x |  |  |  |  |  | 40 | 4 | 65.67 | 13.62 | 0.00 | 0.13 |
| 29 |  |  | x | x |  |  | x |  |  |  | 40 | 5 | 66.44 | 14.39 | 0.00 | 0.15 |
| 19 |  |  | x | x |  |  |  |  |  |  | 40 | 4 | 66.83 | 14.78 | 0.00 | 0.11 |
| 25 |  |  | x |  | x | x |  |  |  |  | 40 | 5 | 66.93 | 14.88 | 0.00 | 0.14 |
| 39 |  |  | x | x |  |  |  | x |  |  | 40 | 5 | 67.52 | 15.48 | 0.00 | 0.13 |
| 27 |  |  | x | x | x |  |  |  |  |  | 40 | 5 | 68.23 | 16.18 | 0.00 | 0.11 |
| 7 |  |  |  |  |  |  | x |  |  |  | 40 | 3 | 69.60 | 17.55 | 0.00 | 0.01 |
| 8 |  |  |  |  | x |  | x |  |  |  | 40 | 4 | 69.68 | 17.63 | 0.00 | 0.04 |
| 26 |  |  | x | x | x | x |  |  |  |  | 40 | 6 | 69.69 | 17.64 | 0.00 | 0.12 |
| 15 |  |  |  |  |  |  | x | x |  |  | 40 | 4 | 70.19 | 18.14 | 0.00 | 0.03 |
| 9 |  |  |  |  | x | x | x |  |  |  | 40 | 5 | 71.63 | 19.58 | 0.00 | 0.03 |

**Table C. General linear model and AICc model selection results for the 27 Wind River iButton temperature sensor sites.** Predictor variables are forage (f), below -5°C (-5), (below -5°C)^2 ­^(-5^2^), below 0°C, (0), (below 0°C)^2^ , above 10°C (A10), (above 10°C)^2^ (A10^2^), total degree days (tdd), (total degree days)^2^ (tdd^2^), below -10°C (-10), (below -10°C)^2 ­^(-10^2^), summer mean (smn), (summer mean) ^2^ (smn^2^), winter mean (wmn), (winter mean) ^2^ (wmn^2^), growing length (gl), (growing length) ^2^ (gl^2^), above 15°C (A15), (above 15°C)^2^ (A15^2^).

| mod | f | -5 | -5^2^ | 0 | 0^2^ | A10 | A10^2^ | tdd | tdd^2^ | -10 | -10^2^ | smn | smn^2^ | wmn | wmn^2^ | gl | gl^2^ | A15 | A15^2^ | n | k | AIC_c_ | ΔAIC_c_ | w_i_ | adj r^2^ |
| --- | --- | --- | --- | --- | --- | --- | --- | --- | --- | --- | --- | --- | --- | --- | --- | --- | --- | --- | --- | --- | --- | --- | --- | --- | --- |
| 1 | x |  |  |  |  | x |  |  |  |  |  |  |  |  |  |  |  |  |  | 27 | 4 | -14.38 | 0.00 | 0.05 | 0.27 |
| 2 |  | x | x |  |  |  |  |  |  |  |  |  |  |  |  |  |  |  |  | 27 | 4 | -13.86 | 0.52 | 0.04 | 0.25 |
| 3 | x | x | x |  |  |  |  |  |  |  |  |  |  |  |  |  |  |  |  | 27 | 5 | -13.53 | 0.84 | 0.03 | 0.29 |
| 4 | x |  |  |  |  | x |  |  |  | x |  |  |  |  |  |  |  |  |  | 27 | 5 | -13.49 | 0.89 | 0.03 | 0.29 |
| 5 | x |  |  |  |  |  |  |  |  |  |  | x |  |  |  |  |  |  |  | 27 | 4 | -13.29 | 1.09 | 0.03 | 0.24 |
| 6 | x | x | x |  |  |  |  | x |  |  |  |  |  |  |  |  |  |  |  | 27 | 6 | -13.24 | 1.14 | 0.03 | 0.34 |
| 7 | x | x |  |  |  | x |  |  |  |  |  |  |  |  |  |  |  |  |  | 27 | 5 | -12.92 | 1.45 | 0.03 | 0.28 |
| 8 |  | x | x |  |  |  |  |  |  |  |  |  |  |  |  | x |  |  |  | 27 | 5 | -12.76 | 1.61 | 0.02 | 0.27 |
| 9 | x | x |  |  |  |  |  | x |  |  |  |  |  |  |  |  |  |  |  | 27 | 5 | -12.68 | 1.70 | 0.02 | 0.27 |
| 10 | x |  |  |  |  | x |  |  |  | x | x |  |  |  |  |  |  |  |  | 27 | 6 | -12.59 | 1.78 | 0.02 | 0.32 |
| 11 | x |  |  |  |  |  |  | x |  |  |  |  |  |  |  |  |  |  |  | 27 | 4 | -12.59 | 1.79 | 0.02 | 0.22 |
| 12 |  | x | x | x |  |  |  |  |  |  |  |  |  |  |  |  |  |  |  | 27 | 5 | -12.53 | 1.85 | 0.02 | 0.27 |
| 13 | x | x | x |  |  |  |  |  |  |  |  |  |  |  |  | x |  |  |  | 27 | 6 | -12.49 | 1.88 | 0.02 | 0.32 |
| 14 | x |  |  |  |  | x |  |  |  |  |  |  |  | x |  |  |  |  |  | 27 | 5 | -12.16 | 2.21 | 0.02 | 0.26 |
| 15 |  | x | x |  |  |  |  | x |  |  |  |  |  |  |  |  |  |  |  | 27 | 5 | -12.16 | 2.22 | 0.02 | 0.26 |
| 16 | x | x | x | x |  |  |  |  |  |  |  |  |  |  |  |  |  |  |  | 27 | 6 | -12.15 | 2.22 | 0.02 | 0.31 |
| 17 | x | x |  |  |  |  |  |  |  |  |  | x |  |  |  |  |  |  |  | 27 | 5 | -12.11 | 2.26 | 0.02 | 0.26 |
| 18 | x |  |  |  |  |  |  |  |  | x |  | x |  |  |  |  |  |  |  | 27 | 5 | -12.00 | 2.37 | 0.02 | 0.25 |
| 19 | x | x | x |  |  | x |  |  |  |  |  |  |  |  |  |  |  |  |  | 27 | 6 | -11.86 | 2.51 | 0.02 | 0.31 |
| 20 | x | x | x |  |  |  |  |  |  |  |  | x |  |  |  |  |  |  |  | 27 | 6 | -11.79 | 2.58 | 0.01 | 0.30 |
| 21 | x |  |  |  |  |  |  | x |  |  |  |  |  | x |  |  |  |  |  | 27 | 5 | -11.70 | 2.68 | 0.01 | 0.24 |
| 22 | x |  |  | x |  |  |  |  |  |  |  |  |  |  |  |  |  |  |  | 27 | 4 | -11.55 | 2.82 | 0.01 | 0.18 |
| 23 | x |  |  |  |  |  |  |  |  |  |  | x |  | x |  |  |  |  |  | 27 | 5 | -11.42 | 2.96 | 0.01 | 0.24 |
| 24 | x |  |  |  |  |  |  |  |  |  |  |  |  |  |  |  |  |  |  | 27 | 5 | -11.40 | 2.97 | 0.01 | 0.24 |
| 25 | x |  |  |  |  | x | x |  |  |  |  |  |  |  |  |  |  |  |  | 27 | 5 | -11.40 | 2.98 | 0.01 | 0.24 |
| 26 | x |  |  |  |  |  |  |  |  |  |  |  |  |  |  | x |  |  |  | 27 | 4 | -11.33 | 3.05 | 0.01 | 0.18 |
| 27 | x |  |  |  |  |  |  |  |  | x |  |  |  |  |  |  |  |  |  | 27 | 4 | -11.14 | 3.23 | 0.01 | 0.17 |
| 28 | x | x | x | x | x |  |  |  |  |  |  |  |  |  |  |  |  |  |  | 27 | 7 | -11.06 | 3.31 | 0.01 | 0.35 |
| 29 | x | x |  | x |  |  |  |  |  |  |  |  |  |  |  |  |  |  |  | 27 | 5 | -11.03 | 3.35 | 0.01 | 0.22 |
| 30 |  | x | x |  |  | x |  |  |  |  |  |  |  |  |  |  |  |  |  | 27 | 5 | -10.99 | 3.39 | 0.01 | 0.22 |
| 31 | x | x |  |  |  |  |  |  |  |  |  |  |  |  |  | x |  |  |  | 27 | 5 | -10.96 | 3.41 | 0.01 | 0.22 |
| 32 |  | x | x |  |  |  |  |  |  |  |  | x |  |  |  |  |  |  |  | 27 | 5 | -10.92 | 3.46 | 0.01 | 0.22 |
| 33 |  | x | x |  |  |  |  |  |  |  |  |  |  |  |  |  |  | x |  | 27 | 5 | -10.86 | 3.52 | 0.01 | 0.22 |
| 34 | x |  |  |  |  |  |  |  |  |  |  |  |  |  |  |  |  | x |  | 27 | 4 | -10.70 | 3.67 | 0.01 | 0.16 |
| 35 | x | x | x |  |  |  |  |  |  |  |  |  |  |  |  |  |  | x |  | 27 | 6 | -10.67 | 3.71 | 0.01 | 0.27 |
| 36 | x |  |  |  |  |  |  | x |  | x |  |  |  |  |  |  |  |  |  | 27 | 5 | -10.64 | 3.74 | 0.01 | 0.21 |
| 37 | x | x |  |  |  |  |  |  |  |  |  |  |  |  |  |  |  |  |  | 27 | 4 | -10.63 | 3.74 | 0.01 | 0.16 |
| mod | f | -5 | -5^2^ | 0 | 0^2^ | A10 | A10^2^ | gdd | gdd^2^ | -10 | -10^2^ | gmn | gmn^2^ | wmn | wmn^2^ | gl | gl^2^ | A15 | A15^2^ | n | k | AIC_c_ | ΔAIC_c_ | w_i_ | adj r^2^ |
| 38 | x |  |  | x |  |  |  |  |  |  |  | x |  |  |  |  |  |  |  | 27 | 5 | -10.58 | 3.80 | 0.01 | 0.21 |
| 39 | x |  |  |  |  |  |  |  |  | x |  |  |  |  |  |  |  | x |  | 27 | 5 | -10.56 | 3.82 | 0.01 | 0.21 |
| 40 | x |  |  |  |  |  |  |  |  |  |  | x |  |  |  | x |  |  |  | 27 | 5 | -10.53 | 3.84 | 0.01 | 0.21 |
| 41 | x |  |  | x |  |  |  |  |  |  |  |  |  | x |  |  |  |  |  | 27 | 5 | -10.46 | 3.91 | 0.01 | 0.21 |
| 42 | x |  |  |  |  |  |  |  |  |  |  | x | x |  |  |  |  |  |  | 27 | 5 | -10.36 | 4.02 | 0.01 | 0.21 |
| 43 |  | x | x | x | x |  |  |  |  |  |  |  |  |  |  |  |  |  |  | 27 | 6 | -10.35 | 4.03 | 0.01 | 0.27 |
| 44 | x |  |  |  |  | x | x |  |  | x |  |  |  |  |  |  |  |  |  | 27 | 6 | -10.33 | 4.04 | 0.01 | 0.27 |
| 45 | x |  |  |  |  |  |  |  |  |  |  |  |  | x |  |  |  |  |  | 27 | 4 | -10.25 | 4.12 | 0.01 | 0.14 |
| 46 | x |  |  |  |  |  |  |  |  |  |  |  |  | x |  | x |  |  |  | 27 | 5 | -10.17 | 4.20 | 0.01 | 0.20 |
| 47 | x | x |  |  |  | x | x |  |  |  |  |  |  |  |  |  |  |  |  | 27 | 6 | -10.13 | 4.25 | 0.01 | 0.21 |
| 48 | x |  |  |  |  |  |  | x |  |  |  |  |  | x | x |  |  |  |  | 27 | 6 | -10.08 | 4.29 | 0.01 | 0.26 |
| 49 | x |  |  |  |  |  |  |  |  | x | x | x |  |  |  |  |  |  |  | 27 | 6 | -9.95 | 4.42 | 0.01 | 0.26 |
| 50 | x | x | x |  |  |  |  |  |  |  |  |  |  |  |  | x | x |  |  | 27 | 7 | -9.94 | 4.44 | 0.01 | 0.32 |
| 51 | x | x |  |  |  |  |  |  |  |  |  | x | x |  |  |  |  |  |  | 27 | 6 | -9.86 | 4.51 | 0.01 | 0.25 |
| 52 |  | x | x |  |  |  |  |  |  |  |  |  |  |  |  | x | x |  |  | 27 | 6 | -9.84 | 4.53 | 0.01 | 0.25 |
| 53 | x | x | x |  |  |  |  | x | x |  |  |  |  |  |  |  |  |  |  | 27 | 7 | -9.80 | 4.57 | 0.01 | 0.32 |
| 54 | x |  |  | x |  |  |  |  |  |  |  |  |  | x | x |  |  |  |  | 27 | 6 | -9.76 | 4.61 | 0.01 | 0.25 |
| 55 | x |  |  |  |  |  |  | x | x |  |  |  |  |  |  |  |  |  |  | 27 | 5 | -9.56 | 4.82 | 0.00 | 0.18 |
| 56 | x |  |  |  |  | x |  |  |  |  |  |  |  | x | x |  |  |  |  | 27 | 6 | -9.49 | 4.89 | 0.00 | 0.24 |
| 57 | x |  |  | x |  |  |  |  |  | x |  |  |  |  |  |  |  |  |  | 27 | 5 | -9.48 | 4.90 | 0.00 | 0.18 |
| 58 | x | x |  |  |  |  |  | x | x |  |  |  |  |  |  |  |  |  |  | 27 | 6 | -9.35 | 5.03 | 0.00 | 0.24 |
| 59 | x |  |  |  |  |  |  |  |  |  |  |  |  | x | x | x |  |  |  | 27 | 6 | -9.29 | 5.09 | 0.00 | 0.24 |
| 60 | x |  |  |  |  |  |  |  |  |  |  |  |  | x | x |  |  |  |  | 27 | 5 | -9.28 | 5.09 | 0.00 | 0.17 |
| 61 | x |  |  |  |  |  |  |  |  | x |  |  |  |  |  | x |  |  |  | 27 | 5 | -9.28 | 5.10 | 0.00 | 0.17 |
| 62 | x |  |  |  |  | x | x |  |  |  |  |  |  | x |  |  |  |  |  | 27 | 6 | -9.25 | 5.13 | 0.00 | 0.23 |
| 63 | x |  |  |  |  | x | x |  |  | x | x |  |  |  |  |  |  |  |  | 27 | 7 | -9.06 | 5.32 | 0.00 | 0.30 |
| 64 |  | x | x |  |  |  |  | x | x |  |  |  |  |  |  |  |  |  |  | 27 | 6 | -9.04 | 5.33 | 0.00 | 0.23 |
| 65 | x |  |  |  |  |  |  |  |  |  |  | x |  | x | x |  |  |  |  | 27 | 6 | -8.96 | 5.42 | 0.00 | 0.23 |
| 66 |  |  |  |  |  |  |  |  |  | x |  |  |  |  |  |  |  |  |  | 27 | 3 | -8.95 | 5.42 | 0.00 | 0.05 |
| 67 | x |  |  | x |  |  |  |  |  |  |  |  |  |  |  |  |  | x |  | 27 | 5 | -8.89 | 5.48 | 0.00 | 0.16 |
| 68 |  |  |  | x |  |  |  |  |  |  |  |  |  | x | x |  |  |  |  | 27 | 5 | -8.81 | 5.57 | 0.00 | 0.16 |
| 69 | x | x |  | x | x |  |  |  |  |  |  |  |  |  |  |  |  |  |  | 27 | 6 | -8.80 | 5.58 | 0.00 | 0.22 |
| 70 |  |  |  |  |  | x |  |  |  | x |  |  |  |  |  |  |  |  |  | 27 | 4 | -8.78 | 5.60 | 0.00 | 0.10 |
| 71 | x |  |  |  |  |  |  |  |  |  |  | x | x | x |  |  |  |  |  | 27 | 6 | -8.77 | 5.60 | 0.00 | 0.22 |
| 72 | x |  |  |  |  |  |  |  |  |  |  |  |  |  |  | x |  | x |  | 27 | 5 | -8.75 | 5.63 | 0.00 | 0.16 |
| 73 | x | x |  |  |  |  |  |  |  |  |  |  |  |  |  |  |  | x | x | 27 | 6 | -8.66 | 5.71 | 0.00 | 0.22 |
| 74 | x |  |  |  |  |  |  |  |  |  |  |  |  |  |  | x | x |  |  | 27 | 5 | -8.65 | 5.73 | 0.00 | 0.15 |
| 75 | x |  |  | x | x |  |  |  |  |  |  |  |  |  |  |  |  |  |  | 27 | 5 | -8.62 | 5.75 | 0.00 | 0.15 |
| 76 |  |  |  | x |  |  |  |  |  |  |  |  |  |  |  |  |  |  |  | 27 | 3 | -8.51 | 5.87 | 0.00 | 0.03 |
| 77 | x |  |  |  |  | x |  |  |  |  |  |  |  |  |  | x | x |  |  | 27 | 6 | -8.50 | 5.88 | 0.00 | 0.21 |
| 78 | x | x |  |  |  |  |  |  |  |  |  |  |  |  |  | x | x |  |  | 27 | 6 | -8.47 | 5.90 | 0.00 | 0.21 |
| 79 |  |  |  |  |  | x |  |  |  |  |  |  |  |  |  |  |  |  |  | 27 | 3 | -8.45 | 5.92 | 0.00 | 0.03 |
| 80 | x |  |  |  |  |  |  | x | x |  |  |  |  | x |  |  |  |  |  | 27 | 6 | -8.40 | 5.97 | 0.00 | 0.21 |
| 81 | x | x |  |  |  |  |  |  |  |  |  |  |  |  |  |  |  | x |  | 27 | 5 | -8.35 | 6.03 | 0.00 | 0.14 |
| mod | f | -5 | -5^2^ | 0 | 0^2^ | A10 | A10^2^ | gdd | gdd^2^ | -10 | -10^2^ | gmn | gmn^2^ | wmn | wmn^2^ | gl | gl^2^ | A15 | A15^2^ | n | k | AIC_c_ | ΔAIC_c_ | w_i_ | adj r^2^ |
| 82 | x | x | x |  |  | x | x |  |  |  |  |  |  |  |  |  |  |  |  | 27 | 7 | -8.26 | 6.12 | 0.00 | 0.28 |
| 83 |  | x | x |  |  |  |  |  |  |  |  |  |  |  |  |  |  | x | x | 27 | 6 | -8.25 | 6.13 | 0.00 | 0.21 |
| 84 |  | x |  | x |  |  |  |  |  |  |  |  |  |  |  |  |  |  |  | 27 | 4 | -8.23 | 6.15 | 0.00 | 0.08 |
| 85 | x |  |  |  |  |  |  | x |  | x | x |  |  |  |  |  |  |  |  | 27 | 6 | -8.23 | 6.15 | 0.00 | 0.21 |
| 86 | x | x | x |  |  |  |  |  |  |  |  | x | x |  |  |  |  |  |  | 27 | 7 | -8.21 | 6.17 | 0.00 | 0.27 |
| 87 | x |  |  |  |  |  |  |  |  | x | x |  |  |  |  |  |  |  |  | 27 | 5 | -8.15 | 6.22 | 0.00 | 0.14 |
| 88 | x |  |  |  |  | x | x |  |  |  |  |  |  |  |  | x |  |  |  | 27 | 6 | -8.11 | 6.27 | 0.00 | 0.20 |
| 89 |  |  |  |  |  |  |  |  |  |  |  |  |  |  |  | x |  |  |  | 27 | 3 | -8.09 | 6.29 | 0.00 | 0.01 |
| 90 |  |  |  |  |  |  |  |  |  |  |  |  |  | x | x | x |  |  |  | 27 | 5 | -8.02 | 6.35 | 0.00 | 0.13 |
| 91 | x |  |  |  |  |  |  |  |  |  |  |  |  | x |  |  |  | x |  | 27 | 5 | -8.01 | 6.37 | 0.00 | 0.13 |
| 92 |  |  |  |  |  |  |  |  |  |  |  |  |  | x | x |  |  |  |  | 27 | 4 | -7.99 | 6.39 | 0.00 | 0.07 |
| 93 |  |  |  |  |  |  |  | x |  |  |  |  |  |  |  |  |  |  |  | 27 | 3 | -7.87 | 6.50 | 0.00 | 0.01 |
| 94 | x |  |  |  |  |  |  |  |  |  |  |  |  |  |  |  |  | x | x | 27 | 5 | -7.87 | 6.50 | 0.00 | 0.13 |
| 95 |  | x |  |  |  |  |  |  |  |  |  |  |  |  |  | x |  |  |  | 27 | 4 | -7.85 | 6.52 | 0.00 | 0.07 |
| 96 | x |  |  |  |  |  |  |  |  |  |  | x |  |  |  | x | x |  |  | 27 | 6 | -7.78 | 6.60 | 0.00 | 0.19 |
| 97 | x |  |  |  |  |  |  |  |  | x | x |  |  |  |  |  |  | x |  | 27 | 6 | -7.76 | 6.61 | 0.00 | 0.19 |
| 98 |  |  |  |  |  | x |  |  |  | x | x |  |  |  |  |  |  |  |  | 27 | 5 | -7.72 | 6.66 | 0.00 | 0.12 |
| 99 | x | x | x |  |  |  |  |  |  |  |  |  |  |  |  |  |  | x | x | 27 | 7 | -7.69 | 6.68 | 0.00 | 0.26 |
| 100 | x |  |  | x | x |  |  |  |  |  |  |  |  | x |  |  |  |  |  | 27 | 6 | -7.65 | 6.72 | 0.00 | 0.19 |
| 101 |  | x | x |  |  | x | x |  |  |  |  |  |  |  |  |  |  |  |  | 27 | 6 | -7.65 | 6.73 | 0.00 | 0.19 |
| 102 |  | x | x |  |  |  |  |  |  |  |  | x | x |  |  |  |  |  |  | 27 | 6 | -7.59 | 6.78 | 0.00 | 0.19 |
| 103 |  |  |  | x |  |  |  |  |  | x |  |  |  |  |  |  |  |  |  | 27 | 4 | -7.50 | 6.88 | 0.00 | 0.05 |
| 104 |  | x |  |  |  |  |  |  |  |  |  |  |  |  |  |  |  |  |  | 27 | 3 | -7.48 | 6.89 | 0.00 | -0.08 |
| 105 | x |  |  | x | x |  |  |  |  |  |  | x |  |  |  |  |  |  |  | 27 | 6 | -7.47 | 6.91 | 0.00 | 0.18 |
| 106 |  |  |  |  |  |  |  |  |  |  |  | x |  |  |  |  |  |  |  | 27 | 3 | -7.39 | 6.98 | 0.00 | -0.01 |
| 107 | x |  |  |  |  |  |  |  |  | x |  |  |  |  |  |  |  | x | x | 27 | 6 | -7.37 | 7.01 | 0.00 | 0.18 |
| 108 | x |  |  |  |  |  |  |  |  |  |  |  |  | x |  | x | x |  |  | 27 | 6 | -7.35 | 7.03 | 0.00 | 0.18 |
| 109 | x |  |  |  |  |  |  | x | x | x |  |  |  |  |  |  |  |  |  | 27 | 6 | -7.32 | 7.05 | 0.00 | 0.18 |
| 110 |  |  |  | x |  |  |  |  |  |  |  |  |  | x |  |  |  |  |  | 27 | 4 | -7.32 | 7.06 | 0.00 | 0.05 |
| 111 |  |  |  |  |  |  |  |  |  | x |  | x |  |  |  |  |  |  |  | 27 | 4 | -7.30 | 7.08 | 0.00 | 0.05 |
| 112 | x |  |  | x |  |  |  |  |  |  |  | x | x |  |  |  |  |  |  | 27 | 6 | -7.28 | 7.10 | 0.00 | 0.18 |
| 113 |  | x |  |  |  |  |  | x |  |  |  |  |  |  |  |  |  |  |  | 27 | 4 | -7.26 | 7.11 | 0.00 | 0.04 |
| 114 |  |  |  |  |  |  |  | x |  | x |  |  |  |  |  |  |  |  |  | 27 | 4 | -7.26 | 7.11 | 0.00 | 0.04 |
| 115 | x |  |  |  |  |  |  |  |  |  |  | x | x |  |  | x |  |  |  | 27 | 6 | -7.22 | 7.15 | 0.00 | 0.18 |
| 116 |  |  |  |  |  |  |  |  |  | x |  |  |  |  |  |  |  | x |  | 27 | 4 | -7.21 | 7.16 | 0.00 | 0.04 |
| 117 |  |  |  |  |  |  |  |  |  | x |  |  |  |  |  | x |  |  |  | 27 | 4 | -7.15 | 7.23 | 0.00 | 0.04 |
| 118 |  |  |  |  |  |  |  |  |  |  |  |  |  | x |  |  |  |  |  | 27 | 3 | -6.96 | 7.42 | 0.00 | -0.02 |
| 119 |  |  |  |  |  |  |  | x |  |  |  |  |  | x | x |  |  |  |  | 27 | 5 | -6.91 | 7.46 | 0.00 | 0.10 |
| 120 |  | x |  |  |  | x |  |  |  |  |  |  |  |  |  |  |  |  |  | 27 | 4 | -6.91 | 7.47 | 0.00 | 0.03 |
| 121 | x |  |  |  |  |  |  |  |  | x | x | x | x |  |  |  |  |  |  | 27 | 7 | -6.76 | 7.61 | 0.00 | 0.23 |
| 122 |  |  |  |  |  |  |  |  |  |  |  |  |  | x |  | x |  |  |  | 27 | 4 | -6.74 | 7.63 | 0.00 | 0.03 |
| 123 |  |  |  |  |  |  |  |  |  |  |  |  |  |  |  |  |  | x |  | 27 | 3 | -6.73 | 7.65 | 0.00 | -0.04 |
| 124 | x |  |  |  |  |  |  |  |  |  |  |  |  | x | x |  |  | x |  | 27 | 6 | -6.71 | 7.66 | 0.00 | 0.16 |
| 125 | x |  |  | x |  |  |  |  |  | x | x |  |  |  |  |  |  |  |  | 27 | 6 | -6.63 | 7.75 | 0.00 | 0.16 |
| mod | f | -5 | -5^2^ | 0 | 0^2^ | A10 | A10^2^ | gdd | gdd^2^ | -10 | -10^2^ | gmn | gmn^2^ | wmn | wmn^2^ | gl | gl^2^ | A15 | A15^2^ | n | k | AIC_c_ | ΔAIC_c_ | w_i_ | adj r^2^ |
| 126 | x |  |  |  |  |  |  | x | x |  |  |  |  | x | x |  |  |  |  | 27 | 7 | -6.47 | 7.91 | 0.00 | 0.22 |
| 127 | x |  |  |  |  |  |  |  |  | x | x |  |  |  |  | x |  |  |  | 27 | 6 | -6.38 | 8.00 | 0.00 | 0.15 |
| 128 |  |  |  |  |  |  |  |  |  | x | x |  |  |  |  |  |  |  |  | 27 | 4 | -6.36 | 8.01 | 0.00 | 0.01 |
| 129 | x |  |  | x | x |  |  |  |  |  |  |  |  | x | x |  |  |  |  | 27 | 7 | -6.30 | 8.08 | 0.00 | 0.22 |
| 130 |  |  |  |  |  |  |  | x |  |  |  |  |  | x |  |  |  |  |  | 27 | 4 | -6.17 | 8.20 | 0.00 | 0.01 |
| 131 | x |  |  | x | x |  |  |  |  | x |  |  |  |  |  |  |  |  |  | 27 | 6 | -6.14 | 8.23 | 0.00 | 0.14 |
| 132 |  |  |  |  |  | x | x |  |  | x |  |  |  |  |  |  |  |  |  | 27 | 5 | -6.13 | 8.25 | 0.00 | 0.07 |
| 133 |  |  |  |  |  | x |  |  |  |  |  |  |  | x |  |  |  |  |  | 27 | 4 | -6.12 | 8.26 | 0.00 | 0.00 |
| 134 |  |  |  |  |  | x |  |  |  |  |  |  |  |  |  | x |  |  |  | 27 | 4 | -6.10 | 8.27 | 0.00 | 0.00 |
| 135 |  |  |  |  |  |  |  |  |  | x | x |  |  |  |  | x |  |  |  | 27 | 6 | -6.10 | 8.27 | 0.00 | 0.14 |
| 136 | x |  |  |  |  | x | x |  |  |  |  |  |  | x | x |  |  |  |  | 27 | 7 | -6.02 | 8.36 | 0.00 | 0.21 |
| 137 |  |  |  |  |  | x |  |  |  |  |  |  |  | x | x |  |  |  |  | 27 | 5 | -5.94 | 8.44 | 0.00 | 0.06 |
| 138 |  |  |  | x | x |  |  |  |  |  |  |  |  |  |  |  |  |  |  | 27 | 4 | -5.91 | 8.46 | 0.00 | 0.00 |
| 139 |  |  |  | x |  |  |  |  |  |  |  | x |  |  |  |  |  |  |  | 27 | 4 | -5.85 | 8.53 | 0.00 | -0.01 |
| 140 |  | x |  |  |  |  |  |  |  |  |  | x |  |  |  |  |  |  |  | 27 | 4 | -5.84 | 8.54 | 0.00 | -0.01 |
| 141 | x | x |  |  |  |  |  |  |  |  |  |  |  |  |  |  |  | x | x | 27 | 6 | -5.80 | 8.57 | 0.00 | 0.13 |
| 142 | x |  |  |  |  |  |  |  |  |  |  |  |  | x | x | x | x |  |  | 27 | 7 | -5.78 | 8.60 | 0.00 | 0.20 |
| 143 | x |  |  |  |  |  |  |  |  |  |  |  |  |  |  | x | x | x |  | 27 | 6 | -5.77 | 8.60 | 0.00 | 0.13 |
| 144 |  |  |  | x |  |  |  |  |  |  |  |  |  |  |  |  |  | x |  | 27 | 4 | -5.74 | 8.64 | 0.00 | -0.01 |
| 145 |  |  |  |  |  | x | x |  |  |  |  |  |  |  |  |  |  |  |  | 27 | 4 | -5.72 | 8.66 | 0.00 | -0.01 |
| 146 | x |  |  | x | x |  |  |  |  |  |  |  |  |  |  |  |  | x |  | 27 | 6 | -5.68 | 8.70 | 0.00 | 0.13 |
| 147 | x |  |  |  |  |  |  |  |  |  |  | x | x | x | x |  |  |  |  | 27 | 7 | -5.67 | 8.70 | 0.00 | 0.20 |
| 148 | x |  |  | x |  |  |  |  |  |  |  |  |  |  |  |  |  | x | x | 27 | 6 | -5.57 | 8.81 | 0.00 | 0.12 |
| 149 |  |  |  | x | x |  |  |  |  |  |  |  |  | x | x |  |  |  |  | 27 | 6 | -5.55 | 8.82 | 0.00 | 0.12 |
| 150 |  |  |  |  |  |  |  |  |  |  |  | x |  |  |  | x |  |  |  | 27 | 4 | -5.50 | 8.88 | 0.00 | -0.02 |
| 151 | x |  |  |  |  |  |  |  |  |  |  |  |  |  |  | x |  | x | x | 27 | 6 | -5.41 | 8.96 | 0.00 | 0.12 |
| 152 |  |  |  |  |  |  |  |  |  |  |  | x |  | x | x |  |  |  |  | 27 | 5 | -5.40 | 8.97 | 0.00 | 0.04 |
| 153 |  |  |  |  |  |  |  |  |  |  |  |  |  |  |  | x |  | x |  | 27 | 4 | -5.31 | 9.06 | 0.00 | -0.03 |
| 154 |  |  |  |  |  |  |  |  |  |  |  |  |  |  |  | x | x |  |  | 27 | 4 | -5.31 | 9.06 | 0.00 | -0.03 |
| 155 |  |  |  | x |  |  |  |  |  | x | x |  |  |  |  |  |  |  |  | 27 | 5 | -5.29 | 9.09 | 0.00 | 0.04 |
| 156 |  |  |  |  |  |  |  | x | x |  |  |  |  |  |  |  |  |  |  | 27 | 4 | -5.26 | 9.11 | 0.00 | -0.03 |
| 157 |  | x |  | x | x |  |  |  |  |  |  |  |  |  |  |  |  |  |  | 27 | 5 | -5.22 | 9.15 | 0.00 | 0.04 |
| 158 | x |  |  |  |  |  |  |  |  |  |  |  |  | x |  |  |  | x | x | 27 | 6 | -5.22 | 9.16 | 0.00 | 0.11 |
| 159 |  |  |  |  |  |  |  |  |  | x | x | x |  |  |  |  |  |  |  | 27 | 5 | -5.17 | 9.20 | 0.00 | 0.04 |
| 160 |  |  |  |  |  |  |  | x |  | x | x |  |  |  |  |  |  |  |  | 27 | 5 | -5.13 | 9.24 | 0.00 | 0.04 |
| 161 |  |  |  |  |  |  |  |  |  |  |  | x |  | x |  |  |  |  |  | 27 | 4 | -5.11 | 9.26 | 0.00 | -0.03 |
| 162 |  |  |  |  |  |  |  |  |  |  |  |  |  | x | x |  |  | x |  | 27 | 5 | -5.05 | 9.33 | 0.00 | 0.30 |
| 163 | x |  |  |  |  |  |  |  |  |  |  |  |  |  |  |  |  |  |  | 27 | 7 | -4.99 | 9.39 | 0.00 | 0.18 |
| 164 |  |  |  | x | x |  |  |  |  | x |  |  |  |  |  |  |  |  |  | 27 | 5 | -4.97 | 9.41 | 0.00 | 0.03 |
| 165 |  | x |  |  |  |  |  |  |  |  |  |  |  |  |  | x | x |  |  | 27 | 5 | -4.89 | 9.48 | 0.00 | 0.03 |
| 166 |  |  |  |  |  |  |  |  |  | x | x |  |  |  |  | x |  |  |  | 27 | 5 | -4.84 | 9.53 | 0.00 | 0.03 |
| 167 |  |  |  |  |  |  |  |  |  | x | x |  |  |  |  |  |  | x |  | 27 | 5 | -4.81 | 9.57 | 0.00 | 0.02 |
| 168 |  |  |  |  |  |  |  |  |  |  |  |  |  |  |  |  |  |  |  | 27 | 7 | -4.79 | 9.59 | 0.00 | 0.18 |
| 169 |  |  |  |  |  |  |  |  |  |  |  | x | x |  |  |  |  |  |  | 27 | 4 | -4.76 | 9.61 | 0.00 | -0.05 |
| mod | f | -5 | -5^2^ | 0 | 0^2^ | A10 | A10^2^ | gdd | gdd^2^ | -10 | -10^2^ | gmn | gmn^2^ | wmn | wmn^2^ | gl | gl^2^ | A15 | A15^2^ | n | k | AIC_c_ | ΔAIC_c_ | w_i_ | adj r^2^ |
| 170 |  | x |  |  |  |  |  |  |  |  |  |  |  |  |  |  |  | x |  | 27 | 4 | -4.74 | 9.63 | 0.00 | -0.05 |
| 171 |  |  |  |  |  | x | x |  |  | x | x |  |  |  |  |  |  |  |  | 27 | 6 | -4.73 | 9.64 | 0.00 | 0.10 |
| 172 |  |  |  |  |  |  |  |  |  |  |  |  |  | x | x | x | x |  |  | 27 | 6 | -4.72 | 9.66 | 0.00 | 0.10 |
| 173 |  |  |  |  |  |  |  | x | x | x |  |  |  |  |  |  |  |  |  | 27 | 5 | -4.47 | 9.90 | 0.00 | 0.01 |
| 174 |  | x |  |  |  |  |  | x | x |  |  |  |  |  |  |  |  |  |  | 27 | 5 | -4.42 | 9.95 | 0.00 | 0.01 |
| 175 |  |  |  |  |  |  |  |  |  | x |  |  |  |  |  |  |  | x | x | 27 | 5 | -4.36 | 10.01 | 0.00 | 0.01 |
| 176 |  |  |  | x | x |  |  |  |  |  |  |  |  | x |  |  |  |  |  | 27 | 5 | -4.30 | 10.08 | 0.00 | 0.01 |
| 177 | x |  |  |  |  |  |  |  |  | x | x |  |  |  |  |  |  | x | x | 27 | 7 | -4.28 | 10.09 | 0.00 | 0.16 |
| 178 |  |  |  |  |  |  |  |  |  | x |  | x | x |  |  |  |  |  |  | 27 | 5 | -4.26 | 10.11 | 0.00 | 0.01 |
| 179 |  | x |  |  |  | x | x |  |  |  |  |  |  |  |  |  |  |  |  | 27 | 5 | -4.25 | 10.12 | 0.00 | 0.00 |
| 180 |  |  |  |  |  |  |  |  |  |  |  |  |  | x |  |  |  | x |  | 27 | 4 | -4.23 | 10.15 | 0.00 | -0.07 |
| 181 |  |  |  |  |  |  |  |  |  |  |  |  |  |  |  |  |  | x | x | 27 | 4 | -4.18 | 10.20 | 0.00 | -0.07 |
| 182 |  |  |  |  |  |  |  |  |  | x |  |  |  |  |  | x | x |  |  | 27 | 5 | -4.16 | 10.22 | 0.00 | 0.00 |
| 183 | x |  |  |  |  |  |  |  |  |  |  | x | x |  |  | x | x |  |  | 27 | 7 | -4.12 | 10.26 | 0.00 | 0.16 |
| 184 |  |  |  |  |  |  |  | x | x |  |  |  |  | x | x |  |  |  |  | 27 | 6 | -3.89 | 10.49 | 0.00 | 0.07 |
| 185 | x |  |  | x | x |  |  |  |  |  |  | x | x |  |  |  |  |  |  | 27 | 7 | -3.80 | 10.57 | 0.00 | 0.14 |
| 186 |  | x |  |  |  |  |  |  |  |  |  | x | x |  |  |  |  |  |  | 27 | 5 | -3.75 | 10.63 | 0.00 | -0.01 |
| 187 |  |  |  |  |  |  |  |  |  |  |  |  |  | x |  | x | x |  |  | 27 | 5 | -3.71 | 10.67 | 0.00 | -0.02 |
| 188 |  |  |  |  |  |  |  | x | x |  |  |  |  | x |  |  |  |  |  | 27 | 5 | -3.41 | 10.97 | 0.00 | -0.03 |
| 189 | x |  |  |  |  |  |  |  |  |  |  |  |  | x | x |  |  | x | x | 27 | 7 | -3.36 | 11.01 | 0.00 | 0.13 |
| 190 |  |  |  |  |  | x | x |  |  |  |  |  |  | x |  |  |  |  |  | 27 | 5 | -3.32 | 11.06 | 0.00 | -0.03 |
| 191 |  |  |  |  |  | x | x |  |  |  |  |  |  |  |  | x |  |  |  | 27 | 5 | -3.08 | 11.29 | 0.00 | -0.04 |
| 192 |  |  |  |  |  | x |  |  |  |  |  |  |  |  |  | x | x |  |  | 27 | 5 | -3.07 | 11.31 | 0.00 | -0.04 |
| 193 |  |  |  | x | x |  |  |  |  |  |  | x |  |  |  |  |  |  |  | 27 | 5 | -3.00 | 11.37 | 0.00 | -0.04 |
| 194 | x |  |  | x | x |  |  |  |  | x | x |  |  |  |  |  |  |  |  | 27 | 7 | -2.93 | 11.44 | 0.00 | 0.12 |
| 195 |  |  |  | x | x |  |  |  |  |  |  |  |  |  |  |  |  | x |  | 27 | 5 | -2.88 | 11.50 | 0.00 | -0.05 |
| 196 |  |  |  | x |  |  |  |  |  |  |  | x | x |  |  |  |  |  |  | 27 | 5 | -2.82 | 11.55 | 0.00 | -0.05 |
| 197 | x |  |  |  |  |  |  |  |  | x | x |  |  |  |  | x | x |  |  | 27 | 7 | -2.79 | 11.58 | 0.00 | 0.11 |
| 198 |  |  |  | x |  |  |  |  |  |  |  |  |  |  |  |  |  | x | x | 27 | 5 | -2.75 | 11.62 | 0.00 | -0.05 |
| 199 |  |  |  |  |  | x | x |  |  |  |  |  |  | x | x |  |  |  |  | 27 | 6 | -2.62 | 11.76 | 0.00 | 0.02 |
| 200 |  |  |  |  |  |  |  |  |  |  |  | x | x | x |  |  |  |  |  | 27 | 5 | -2.60 | 11.77 | 0.00 | -0.06 |
| 201 |  | x |  |  |  |  |  |  |  |  |  |  |  |  |  |  |  | x | x | 27 | 5 | -2.58 | 11.80 | 0.00 | -0.06 |
| 202 |  |  |  |  |  |  |  | x | x | x | x |  |  |  |  |  |  |  |  | 27 | 6 | -2.51 | 11.86 | 0.00 | 0.02 |
| 203 |  |  |  | x | x |  |  |  |  | x | x |  |  |  |  |  |  |  |  | 27 | 6 | -2.50 | 11.87 | 0.00 | 0.02 |
| 204 |  |  |  |  |  |  |  |  |  |  |  | x | x |  |  | x |  |  |  | 27 | 5 | -2.48 | 11.90 | 0.00 | -0.06 |
| 205 |  |  |  |  |  |  |  |  |  |  |  | x |  |  |  | x | x |  |  | 27 | 5 | -2.46 | 11.91 | 0.00 | -0.06 |
| 206 |  |  |  |  |  |  |  |  |  |  |  |  |  |  |  | x |  | x | x | 27 | 5 | -2.30 | 12.07 | 0.00 | -0.07 |
| 207 | x |  |  |  |  |  |  |  |  |  |  |  |  |  |  | x | x | x | x | 27 | 7 | -2.29 | 12.09 | 0.00 | 0.10 |
| 208 |  |  |  |  |  |  |  |  |  |  |  |  |  |  |  | x | x | x |  | 27 | 5 | -2.27 | 12.10 | 0.00 | -0.07 |
| 209 |  |  |  |  |  |  |  |  |  |  |  | x | x | x | x |  |  |  |  | 27 | 6 | -2.19 | 12.18 | 0.00 | 0.01 |
| 210 |  |  |  |  |  |  |  |  |  | x | x | x | x |  |  |  |  |  |  | 27 | 6 | -2.19 | 12.19 | 0.00 | 0.01 |
| mod | f | -5 | -5^2^ | 0 | 0^2^ | A10 | A10^2^ | gdd | gdd^2^ | -10 | -10^2^ | gmn | gmn^2^ | wmn | wmn^2^ | gl | gl^2^ | A15 | A15^2^ | n | k | AIC_c_ | ΔAIC_c_ | w_i_ | adj r^2^ |
| 211 | x |  |  | x | x |  |  |  |  |  |  |  |  |  |  |  |  | x | x | 27 | 7 | -2.15 | 12.23 | 0.00 | 0.10 |
| 212 |  |  |  |  |  |  |  |  |  |  |  |  |  | x | x |  |  | x | x | 27 | 6 | -1.97 | 12.40 | 0.00 | 0.00 |
| 213 |  |  |  |  |  |  |  |  |  |  |  |  |  | x |  |  |  | x | x | 27 | 5 | -1.73 | 12.65 | 0.00 | -0.09 |
| 214 |  |  |  |  |  |  |  |  |  | x | x |  |  |  |  |  |  | x | x | 27 | 6 | -1.72 | 12.65 | 0.00 | -0.01 |
| 215 |  |  |  |  |  |  |  |  |  | x | x |  |  |  |  | x | x |  |  | 27 | 6 | -1.59 | 12.79 | 0.00 | -0.02 |
| 216 |  |  |  |  |  | x | x |  |  |  |  |  |  |  |  | x | x |  |  | 27 | 6 | 0.26 | 14.64 | 0.00 | -0.09 |
| 217 |  |  |  | x | x |  |  |  |  |  |  | x | x |  |  |  |  |  |  | 27 | 6 | 0.32 | 14.69 | 0.00 | -0.09 |
| 218 |  |  |  | x | x |  |  |  |  |  |  |  |  |  |  |  |  | x | x | 27 | 6 | 0.47 | 14.84 | 0.00 | -0.10 |
| 219 |  |  |  |  |  |  |  |  |  |  |  | x | x |  |  | x | x |  |  | 27 | 6 | 0.87 | 15.24 | 0.00 | -0.11 |
| 220 |  |  |  |  |  |  |  |  |  |  |  |  |  |  |  | x | x | x | x | 27 | 6 | 1.03 | 15.41 | 0.00 | -0.12 |

**Table D. Correlation values for habitat variables in the Winds (2010) in upper right half of the table and for the Bighorns (2011) on the lower left half of the table.**

|  | Elev | ElevSQ | ForPatch | ForPatchSQ | Depth | DepthSQ | Aspect | ForPerim | ForPerimSQ | DiffNearPk |
| --- | --- | --- | --- | --- | --- | --- | --- | --- | --- | --- |
| Elev | **1.00** | 1.00 | 0.08 | 0.08 | -0.21 | -0.24 | 0.15 | -0.41 | -0.29 | -0.59 |
| ElevSQ | 1.00 | **1.00** | 0.08 | 0.08 | -0.21 | -0.25 | 0.16 | -0.41 | -0.30 | -0.59 |
| ForPatch | -0.37 | -0.37 | **1.00** | 0.94 | -0.18 | -0.17 | -0.21 | -0.03 | -0.07 | -0.20 |
| ForPatch  SQ | -0.33 | -0.33 | 0.94 | **1.00** | -0.20 | -0.18 | -0.26 | -0.02 | -0.05 | -0.14 |
| Depth | -0.06 | -0.03 | -0.23 | -0.27 | **1.00** | 0.97 | 0.11 | 0.06 | 0.01 | 0.32 |
| DepthSQ | -0.10 | -0.07 | -0.22 | -0.25 | 0.99 | **1.00** | 0.08 | 0.07 | 0.02 | 0.33 |
| Aspect | -0.15 | -0.13 | 0.15 | 0.16 | 0.03 | 0.05 | **1.00** | 0.08 | 0.12 | -0.06 |
| ForPerim | -0.74 | -0.74 | 0.30 | 0.32 | 0.04 | 0.06 | 0.17 | **1.00** | 0.95 | 0.22 |
| ForPerim  SQ | -0.67 | -0.66 | 0.27 | 0.28 | 0.13 | 0.14 | 0.15 | 0.96 | **1.00** | 0.21 |
| DiffNear  Pk | -0.22 | -0.23 | 0.04 | 0.05 | -0.12 | -0.13 | -0.03 | 0.11 | 0.10 | **1.00** |

**Table E. Correlation values for forage availability and climate variables in the Winds (2010).**

|  | ForagePerim | Below0 | Below-5 | Below-10 | Above10 | Above15 | Winter  Mean | Grow  Length | Summer  Mean | TotalDegree  Days | Elevation |
| --- | --- | --- | --- | --- | --- | --- | --- | --- | --- | --- | --- |
| ForagePatch | -0.27 | -0.07 | 0.04 | -0.21 | -0.21 | -0.24 | 0.03 | 0.04 | -0.30 | -0.15 | 0.07 |
| ForagePerim | **1.00** | 0.05 | -0.38 | -0.22 | 0.00 | 0.01 | 0.25 | -0.05 | 0.17 | 0.07 | -0.41 |
| Below0 |  | **1.00** | 0.36 | 0.18 | -0.49 | -0.26 | -0.41 | -0.96 | -0.41 | -0.87 | 0.46 |
| Below-5 |  |  | **1.00** | 0.54 | -0.11 | 0.10 | -0.93 | -0.41 | -0.18 | -0.37 | 0.44 |
| Below-10 |  |  |  | **1.00** | 0.12 | 0.41 | -0.58 | -0.18 | 0.10 | -0.07 | 0.20 |
| Above10 |  |  |  |  | **1.00** | 0.66 | 0.06 | 0.48 | 0.88 | 0.74 | -0.59 |
| Above15 |  |  |  |  |  | **1.00** | -0.12 | 0.25 | 0.81 | 0.57 | -0.22 |
| WinterMean |  |  |  |  |  |  | **1.00** | 0.42 | 0.15 | 0.35 | -0.29 |
| GrowLength |  |  |  |  |  |  |  | **1.00** | 0.40 | 0.90 | -0.49 |
| SummerMean |  |  |  |  |  |  |  |  | **1.00** | 0.74 | -0.53 |
| TotalDegreeDays |  |  |  |  |  |  |  |  |  | **1.00** | -0.58 |
| Elevation |  |  |  |  |  |  |  |  |  |  | **1.00** |
