# Supplementary material for: Climate Tolerances and Habitat Requirements Jointly Shape the Elevational Distribution of the American Pika (Ochotona princeps), with Implications for Climate Change Effects
Source: PLoS One. 2015 Aug 5;10(8):e0131082. doi: 10.1371/journal.pone.0131082 (PMC4526653; doi:10.1371/journal.pone.0131082)
Supplement: S2 File — (DOCX) [file pone.0131082.s002.docx]

**S2 File.**

**Table A.** Habitat and scat per m^2^ data for each site collected in 2010 for the Wind River Range, WY (n = 43).

| *Site* | *Scat* | *elevation* | *patch forage* | *depth* | *aspect* | *perimeter forage* | *difference to near summit* |
| --- | --- | --- | --- | --- | --- | --- | --- |
| BLD38 | 0.62 | 3206 | 13 | 65 | 307 | 0.13 | 132 |
| BLD39 | 0.37 | 3348 | 43 | 50 | 55 | 0 | 193 |
| BLD40 | 0.12 | 3849 | 39 | 68 | 333 | 0 | 30 |
| BSD10 | 0.18 | 3519 | 13 | 84 | 53 | 0 | 351 |
| BSD13 | 0.49 | 3313 | 13 | 69 | 228 | 0.06 | 225 |
| BSD33 | 0.12 | 3280 | 7 | 103 | 248 | 0.54 | 402 |
| BSD43 | 0.02 | 3752 | 18 | 96 | 234 | 0 | 87 |
| BSD8 | 0.49 | 3518 | 65 | 67 | 24 | 0 | 74 |
| BSD9 | 0.39 | 2995 | 21 | 123 | 125 | 0.29 | 244 |
| CSD41 | 0.63 | 3460 | 57 | 62.5 | 121 | 0.26 | 240 |
| CSD42 | 0.49 | 3262 | 29 | 77 | 163 | 0 | 438 |
| CSD7 | 0.03 | 3373 | 5 | 91 | 212 | 0 | 327 |
| FLD34 | 0.34 | 3240 | 9 | 70 | 175 | 0 | 269 |
| NFL25 | 0.05 | 3228 | 9 | 92 | 105 | 0.38 | 142 |
| NFL26 | 0.09 | 3486 | 3 | 64 | 257 | 0.62 | 66 |
| NFL27 | 0.17 | 3214 | 0 | 72 | 213 | 0.23 | 236 |
| NFL29 | 0.55 | 3250 | 27 | 100 | 328 | 0.59 | 428 |
| NFL30 | 0.26 | 3616 | 12 | 76 | 238 | 0 | 62 |
| NFL31 | 0.52 | 3536 | 24 | 71 | 147 | 0 | 75 |
| NFL32 | 0.37 | 3554 | 18 | 45 | 285 | 0 | 57 |
| NFL35 | 0.26 | 3037 | 9 | 48 | 64 | 0 | 250 |
| SCD11 | 0.15 | 3646 | 21 | 66 | 159 | 0 | 112 |
| SCD12 | 0.22 | 3707 | 35 | 69 | 275 | 0 | 51 |
| SCD14 | 0.13 | 3276 | 7 | 99 | 290 | 0.15 | 241 |
| SCD15 | 0.07 | 3716 | 4 | 85 | 222 | 0 | 76 |
| SCD16 | 0.6 | 3503 | 12 | 75 | 132 | 0 | 297 |
| TLD1 | 0.15 | 2736 | 1 | 75 | 297 | 0 | 501 |
| TLD2 | 0.2 | 3211 | 22 | 96 | 33 | 0 | 106 |
| TLD4 | 0.22 | 3564 | 17 | 100 | 250 | 0 | 55 |
| TLD5 | 0.53 | 3390 | 14 | 81.4 | 116 | 0.17 | 171 |
| TLD6 | 0.08 | 3503 | 20 | 100 | 337 | 0 | 116 |
| WM0 | 0.31 | 2948 | 21 | 32 | 157 | 0.55 | 161 |
| WM17 | 0.34 | 3249 | 21 | 69 | 34 | 0.27 | 91 |
| WM18 | 0.31 | 3447 | 24 | 72 | 145 | 0 | 53 |
| WM19 | 0.31 | 3439 | 13 | 82 | 293 | 0.08 | 28 |
| WM21 | 0.08 | 3289 | 9 | 75 | 111 | 0 | 234 |
| WM22 | 0.16 | 3470 | 25 | 92 | 247 | 0 | 52 |
| WM23 | 0.15 | 3431 | 23 | 66 | 329 | 0.06 | 132 |
| WM24 | 0.37 | 3272 | 36 | 67 | 260 | 0.24 | 116 |
| WRP20 | 0.07 | 3913 | 13 | 57 | 288 | 0 | 105 |
| WRP28 | 0.04 | 3926 | 6 | 62.5 | 148 | 0 | 92 |
| WRP36 | 0.16 | 3092 | 10 | 169 | 205 | 0.06 | 368 |
| WRP37 | 0.33 | 2540 | 34 | 97 | 209 | 0.35 | 308 |

**Table B.** Habitat and scat per m^2^ data for each site collected in 2011 for the Bighorn Range (n = 40).

| *Site* | *Scat* | *elevation* | *patch forage* | *depth* | *aspect* | *perimeter forage* | *difference to near summit* | |
| --- | --- | --- | --- | --- | --- | --- | --- | --- |
| BH1 | 0.63 | 2701 | 19 | 44.5 | 170 | 0.48 | 79 |  |
| BH10 | 0.66 | 2923 | 8 | 56.44 | 235 | 0.16 | 240 |  |
| BH11 | 0.12 | 2857 | 31 | 88.35 | 199 | 0 | 119 |  |
| BH12 | 0.63 | 2966 | 5 | 56.67 | 29 | 0.16 | 234 |  |
| BH13 | 0.38 | 3517 | 7 | 95.45 | 156 | 0 | 56 |  |
| BH14 | 0.52 | 3234 | 11 | 76.41 | 41 | 0 | 192 |  |
| BH15 | 0.1 | 2633 | 4 | 96 | 276 | 0.49 | 8 |  |
| BH16 | 0.25 | 2618 | 13 | 78.08 | 193 | 0.87 | 65 |  |
| BH17 | 0.47 | 3550 | 13 | 68.7 | 53 | 0 | 12 |  |
| BH18 | 0.14 | 3700 | 3 | 95.95 | 252 | 0 | 33 |  |
| BH19 | 0.38 | 3467 | 14 | 82.1 | 196 | 0 | 23 |  |
| BH2 | 0.78 | 3015 | 30 | 63.66 | 290 | 0.37 | 71 |  |
| BH20 | 0.51 | 3588 | 9 | 57.4 | 147 | 0 | 92 |  |
| BH21 | 1.91 | 2682 | 17 | 77.85 | 284 | 0.55 | 288 |  |
| BH22 | 0.11 | 3754 | 3 | 82.65 | 125 | 0 | 26 |  |
| BH23 | 0.06 | 3736 | 12 | 52.1 | 236 | 0 | 22 |  |
| BH24 | 0.2 | 3443 | 2 | 59.48 | 278 | 0 | 130 |  |
| BH25 | 0.44 | 3288 | 13 | 46.5 | 319 | 0 | 75 |  |
| BH26 | 0 | 3897 | 6 | 66.2 | 161 | 0 | 13 |  |
| BH27 | 0.46 | 3541 | 21 | 43.3 | 257 | 0 | 236 |  |
| BH28 | 0.7 | 3209 | 13 | 62.67 | 108 | 0 | 298 |  |
| BH29 | 0.55 | 3165 | 24 | 66.45 | 249 | 0.45 | 30 |  |
| BH3 | 1.48 | 2776 | 6 | 80.65 | 144 | 0.62 | 103 |  |
| BH30 | 1.73 | 3086 | 7 | 61.85 | 129 | 0.65 | 199 |  |
| BH31 | 0.86 | 3228 | 13 | 64.91 | 74 | 0.33 | 66 |  |
| BH32 | 1.04 | 3143 | 5 | 62.5 | 318 | 0.27 | 60 |  |
| BH33 | 0 | 3714 | 8 | 76.44 | 48 | 0 | 80 |  |
| BH34 | 0.34 | 3570 | 16 | 67.55 | 122 | 0 | 224 |  |
| BH35 | 1.81 | 3122 | 40 | 56.58 | 105 | 0.63 | 99 |  |
| BH36 | 1.55 | 2742 | 26 | 77.3 | 39 | 0 | 38 |  |
| BH37 | 0.12 | 3573 | 2 | 58.81 | 239 | 0 | 8 |  |
| BH38 | 0.08 | 3720 | 4 | 72.18 | 211 | 0 | 300 |  |
| BH39 | 0.1 | 3640 | 11 | 59.58 | 232 | 0 | 40 |  |
| BH4 | 0 | 2158 | 22 | 96.48 | 330 | 0.69 | 179 |  |
| BH40 | 0.35 | 3365 | 29 | 71.44 | 260 | 0 | 95 |  |
| BH5 | 0.96 | 2753 | 52 | 36.85 | 286 | 0.58 | 172 |  |
| BH6 | 0.63 | 2969 | 17 | 53 | 208 | 0.19 | 98 |  |
| BH7 | 0.55 | 2980 | 2 | 26.61 | 75 | 0.13 | 99 |  |
| BH8 | 1.79 | 2901 | 33 | 46.62 | 136 | 0.47 | 83 |  |
| BH9 | 0.33 | 2955 | 3 | 81.6 | 149 | 0.56 | 204 |  |
