# Supplementary material for: Climate Tolerances and Habitat Requirements Jointly Shape the Elevational Distribution of the American Pika (Ochotona princeps), with Implications for Climate Change Effects
Source: PLoS One. 2015 Aug 5;10(8):e0131082. doi: 10.1371/journal.pone.0131082 (PMC4526653; doi:10.1371/journal.pone.0131082)
Supplement: S3 File — (DOCX) [file pone.0131082.s003.docx]

**S3 File.**

**Table A.** Climate variables derived from temperature-sensors data collected 19 September 2010 through 17 August 2011 for sites in the Wind River Range (n = 27). Variables by site are scat per m^2^ (S), days below 0°C (0), days below -5°C (-5), days below -10°C (-10), days above 10°C (A10), days above 15°C (A15)**,** winter mean temperature (wmn), growing length (gl), summer mean temperature (smn), total degree days (tdd).

| Site | S | 0 | -5 | -10 | A10 | A15 | wmn | gl | smn | tdd |
| --- | --- | --- | --- | --- | --- | --- | --- | --- | --- | --- |
| SCD11 | 0.15 | 225 | 0 | 0 | 8 | 0 | -1.64 | 83 | 8.01 | 665.12 |
| SCD12 | 0.22 | 225 | 0 | 0 | 8 | 0 | -1.64 | 83 | 8.01 | 665.12 |
| BSD13 | 0.49 | 199 | 3 | 0 | 45 | 24 | -2.44 | 109 | 13.00 | 1417.00 |
| SCD14 | 0.13 | 284 | 0 | 0 | 13 | 0 | -3.35 | 60 | 8.88 | 532.86 |
| SCD16 | 0.6 | 242 | 112 | 0 | 35 | 1 | -4.75 | 63 | 10.44 | 657.64 |
| WM17 | 0.34 | 257 | 52 | 0 | 33 | 0 | -3.44 | 50 | 9.52 | 476.06 |
| WM18 | 0.31 | 209 | 70 | 0 | 27 | 0 | -4.22 | 109 | 8.75 | 954.25 |
| WM22 | 0.16 | 249 | 77 | 0 | 38 | 0 | -3.95 | 56 | 10.69 | 598.69 |
| WM23 | 0.15 | 227 | 9 | 0 | 32 | 0 | -2.99 | 78 | 8.97 | 699.46 |
| NFL25 | 0.05 | 211 | 46 | 0 | 43 | 22 | -4.17 | 97 | 13.44 | 1303.68 |
| NFL26 | 0.09 | 284 | 6 | 0 | 3 | 0 | -3.67 | 21 | 7.58 | 159.16 |
| NFL27 | 0.17 | 200 | 1 | 0 | 28 | 3 | -1.03 | 108 | 10.21 | 1102.95 |
| WRP28 | 0.04 | 272 | 222 | 19 | 0 | 0 | -7.00 | 43 | 5.25 | 225.75 |
| TLD2 | 0.2 | 246 | 33 | 0 | 39 | 0 | -3.51 | 61 | 9.57 | 583.64 |
| NFL30 | 0.26 | 252 | 163 | 0 | 20 | 0 | -5.17 | 54 | 8.06 | 435.50 |
| NFL31 | 0.52 | 235 | 137 | 0 | 16 | 0 | -5.14 | 75 | 7.97 | 597.70 |
| NFL32 | 0.37 | 246 | 171 | 5 | 2 | 0 | -5.99 | 58 | 7.32 | 424.51 |
| BSD33 | 0.12 | 207 | 0 | 0 | 22 | 0 | -2.62 | 104 | 10.02 | 1041.63 |
| BLD38 | 0.62 | 206 | 122 | 1 | 47 | 0 | -5.37 | 107 | 10.13 | 1084.44 |
| BLD39 | 0.37 | 225 | 60 | 0 | 0 | 0 | -4.02 | 79 | 6.12 | 483.66 |
| CSD41 | 0.63 | 257 | 157 | 6 | 11 | 0 | -5.21 | 56 | 7.77 | 435.14 |
| CSD42 | 0.49 | 185 | 40 | 0 | 53 | 18 | -3.53 | 130 | 11.31 | 1470.52 |
| TLD4 | 0.22 | 252 | 179 | 39 | 13 | 0 | -6.74 | 57 | 8.29 | 472.29 |
| TLD5 | 0.53 | 247 | 154 | 6 | 50 | 34 | -5.24 | 57 | 14.55 | 829.50 |
| TLD6 | 0.08 | 252 | 159 | 5 | 4 | 0 | -5.71 | 56 | 7.27 | 406.89 |
| CSD7 | 0.03 | 235 | 177 | 62 | 55 | 34 | -6.59 | 76 | 12.53 | 952.53 |
| BSD9 | 0.39 | 207 | 50 | 0 | 34 | 0 | -4.27 | 101 | 9.31 | 940.77 |
